# DSC MRI in the human brain using deoxyhemoglobin and gadolinium—Simulations and validations at 3T

**DOI:** 10.3389/fnimg.2023.1048652

**Published:** 2023-02-20

**Authors:** Jacob Benjamin Schulman, Ece Su Sayin, Angelica Manalac, Julien Poublanc, Olivia Sobczyk, James Duffin, Joseph A. Fisher, David Mikulis, Kâmil Uludağ

**Affiliations:** ^1^Department of Medical Biophysics, University of Toronto, Toronto, ON, Canada; ^2^Krembil Brain Institute, University Health Network, Toronto, ON, Canada; ^3^Department of Physiology, University of Toronto, Toronto, ON, Canada; ^4^Joint Department of Medical Imaging and the Functional Neuroimaging Lab, University Health Network, Toronto, ON, Canada; ^5^Department of Anaesthesia and Pain Management, University Health Network, University of Toronto, Toronto, ON, Canada; ^6^Toronto General Hospital Research Institute, Toronto General Hospital, Toronto, ON, Canada; ^7^The Joint Department of Medical Imaging, The Toronto Western Hospital, Toronto, ON, Canada; ^8^Center for Neuroscience Imaging Research, Institute for Basic Science & Department of Biomedical Engineering, Sungkyunkwan University, Suwon, Republic of Korea

**Keywords:** perfusion, DSC, gadolinium, simulations, deoxyhemoglobin

## Abstract

**Introduction:**

Dynamic susceptibility contrast (DSC) MRI allows clinicians to determine perfusion parameters in the brain, such as cerebral blood flow, cerebral blood volume, and mean transit time. To enable quantification, susceptibility changes can be induced using gadolinium (Gd) or deoxyhemoglobin (dOHb), the latter just recently introduced as a contrast agent in DSC. Previous investigations found that experimental parameters and analysis choices, such as the susceptibility amplitude and partial volume, affect perfusion quantification. However, the accuracy and precision of DSC MRI has not been systematically investigated, particularly in the lower susceptibility range.

**Methods:**

In this study, we compared perfusion values determined using Gd with values determined using a contrast agent with a lower susceptibility—dOHb—under different physiological conditions, such as varying the baseline blood oxygenation and/or magnitude of hypoxic bolus, by utilizing numerical simulations and conducting experiments on healthy subjects at 3T. The simulation framework we developed for DSC incorporates MRI signal contributions from intravascular and extravascular proton spins in arterial, venous, and cerebral tissue voxels. This framework allowed us to model the MRI signal in response to both Gd and dOHb.

**Results and discussion:**

We found, both in the experimental results and simulations, that a reduced intravascular volume of the selected arterial voxel, reduced baseline oxygen saturation, greater susceptibility of applied contrast agent (Gd vs. dOHb), and/or larger magnitude of applied hypoxic bolus reduces the overestimation and increases precision of cerebral blood volume and flow. As well, we found that normalizing tissue to venous rather than arterial signal increases the accuracy of perfusion quantification across experimental paradigms. Furthermore, we found that shortening the bolus duration increases the accuracy and reduces the calculated values of mean transit time. In summary, we experimentally uncovered an array of perfusion quantification dependencies, which agreed with the simulation framework predictions, using a wider range of susceptibility values than previously investigated. We argue for caution when comparing absolute and relative perfusion values within and across subjects obtained from a standard DSC MRI analysis, particularly when employing different experimental paradigms and contrast agents.

## Introduction

Cerebral perfusion imaging is used to investigate diseases characterized by vascular abnormality, such as atherosclerotic disease, vasculitis, Moyamoya disease, and neoplasms (Harris et al., 1998; Law et al., 2008; Copen et al., 2011; Qiao et al., 2017). In dynamic susceptibility contrast (DSC) MRI, a bolus of paramagnetic contrast agent is tracked as it passes through the vasculature, enabling perfusion quantification based on the principles of indicator dilution theory (Zierler, 1962; Rosen et al., 1990). The paramagnetic contrast agent creates inhomogeneities in the local magnetic field, reducing signal by diminishing the phase coherence and increasing the relaxation rate ($R_{2}^{*}$) of extravascular and intravascular water proton spins (Rosen et al., 1990). The signal time courses resulting from the passage of contrast agent are then used to calculate cerebral blood volume (*CBV*), cerebral blood flow (*CBF*) and mean transit time (*MTT*), all of which possessing clinical and research utility (Harris et al., 1998; Law et al., 2008; Copen et al., 2011; Tanaka et al., 2011; Wang et al., 2013; Qiao et al., 2017).

Gadolinium (Gd), a paramagnetic metal administered intravenously, is the standard contrast agent used in DSC MRI (Jahng et al., 2014). It has long been known that deoxyhemoglobin (dOHb), the deoxygenated form of oxygen's carrier protein in our blood, hemoglobin, is paramagnetic as well (Pauling and Coryell, 1936). While being used to localize neuronal activity in functional MRI (fMRI) since the early 1990s (Ogawa et al., 1990, 1992; Bandettini et al., 1992; Kwong et al., 1992), the paramagnetic properties of dOHb were only recently exploited in humans to generate a bolus for DSC MRI analysis by transiently reducing the oxygen concentration in inhaled gas (Poublanc et al., 2021; Vu et al., 2021; Lee et al., 2022). Although not using a standard DSC analysis, it should be noted that hypoxia was used to estimate *CBV* in cats as early as 1998 (van Zijl et al., 1998).

A critical area of DSC MRI research pertains to modeling the changes in $R_{2}^{*}$ that result from the passage of contrast agent through simulated vasculature (Weisskoff et al., 1994; Boxerman et al., 1995; van Zijl et al., 1998). By incorporating the effects of contrast agent susceptibility and tissue structure, researchers have been able to simulate and understand how various properties, such as vessel heterogeneity, tissue composition, and contrast agent extraversion, affect DSC MRI signal and perfusion quantification (Kiselev, 2001; Kjølby et al., 2009; Quarles et al., 2009; Semmineh et al., 2014; Digernes et al., 2017). These simulation studies have so far been limited to DSC MRI applications when using Gd.

From a simulation standpoint, Gd and dOHb can be modeled with the same framework for DSC MRI, with the only major differences between them being bolus shape, vascular compartmentalization, and molar susceptibility: Gd induces a higher susceptibility change than dOHb; Gd is limited to the plasma fraction whereas dOHb is limited to the red blood cell fraction; currently, the dOHb bolus can only be generated to be longer in duration than the Gd bolus. While researchers have investigated the accuracy and precision of perfusion quantification in the higher susceptibility range (references in Calamante, 2013), this has not been investigated in the lower susceptibility range. Some of the parameters relevant to perfusion quantification include the dosage of contrast agent [in the case of dOHb, the magnitude of the hypoxic bolus (ΔS_a_O_2_)], baseline oxygen saturation (base-S_a_O_2_), bolus duration, and choice of reference voxel (i.e., artery or vein).

To examine these questions, we developed a DSC MRI signal model, which incorporates separate vascular compartments with both linear extravascular and quadratic intravascular relaxation rate contributions, as used in a previous fMRI model (Uludag et al., 2009). This enabled us to model tissue, arterial, and venous signal in response to generic contrast agents with arbitrary susceptibility values and bolus characteristics, such as duration and shape. We use this model to examine the effects of contrast agent susceptibility, ΔS_a_O_2_, base-S_a_O_2_, bolus duration, tissue properties, and the choice of reference voxel on perfusion quantification. This framework is theoretically applicable to any contrast agent if its molar susceptibility is known. Experimentally, bolus properties such as ΔS_a_O_2_, base-S_a_O_2_, and duration were easily controllable with the gas control system used to create dOHb contrast, allowing us to study the effects of these parameters. We validated our model by comparing the simulation predictions to experimental results obtained from six healthy subjects at 3T when using dOHb or Gd as contrast.

## DSC MRI signal theory

### Section 1: Forward model

The forward model is based on work by Uludag et al., 2009, originally developed to model gradient- (GRE) and spin-echo (SE) fMRI signals for magnetic field strengths between 1.5 and 16.4T (Uludag et al., 2009). As the fMRI signal change results from susceptibility variations, caused by dOHb in the case of neuronal activation or vascular challenges, the model can also be applied to DSC MRI utilizing any contrast agent that induces magnetic susceptibility changes. Here, this framework is generalized and applied to model 3T GRE signal changes induced by boluses of dOHb and Gd (please see the Discussion section for other DSC signal models and how they relate to the proposed model).

#### Signal intensity

${\text{T}}_{2}^{*}$-weighted MRI signal (*S*_*tot*_) is composed of extravascular (*EES*) and intravascular (*IVS*) signal contributions, weighted by their respective volumes (Uludag et al., 2009):^1^


(1) $$\begin{array}{l} S_{tot}=\;{\left(1-CBV\right)\cdot S}_{EES}+\underset{i}{\sum }{CBV_{i}\cdot S}_{IVS,\;i} \end{array}$$

*S*_*EES*_ and *S*_*IVS*_ are the extravascular and intravascular signal contributions^2^, respectively. The index *i* denotes the specific vascular compartment within the voxel being simulated (artery, vein, capillary, venule, or arteriole). Therefore, the signal is a summation of extravascular signal along with various intravascular signal contributions, each scaled by their volume proportion within the voxel. Of note, many DSC signal models from previous clinical research studies do not distinguish *S*_*IVS*_ and *S*_*EES*_ components (Calamante et al., 2000; Patil and Johnson, 2011; Patil et al., 2013; Chappell et al., 2015). The separate signal components can be written as:


(2) $$\begin{array}{l} S_{EES}=e^{-TE\cdot \left(R_{2,0,EES}^{*}+R_{2,con,EES}^{*}\right)} \end{array}$$

(3) $$\begin{array}{l} S_{IVS}=e^{-TE\cdot \left(R_{2,0,IVS}^{*}+R_{2,con,IVS}^{*}\right)} \end{array}$$

*S*_*EES*_ and *S*_*IVS*_ (Eqs. 2 and 3) are modeled as mono exponentials to the power of the negative echo time (here, TE is set to 30 ms) multiplied by the transverse relaxation rate ($R_{2}^{*}$), the inverse of the transverse relaxation time (Yablonskiy and Haacke, 1994). $R_{2,con,EES}^{*}$ and $R_{2,con,IVS}^{*}$ are the magnetic relaxation rates induced by applied contrast agent in the extravascular and intravascular space of a voxel, respectively. $R_{2,0,EES}^{*}$ and $R_{2,0,IVS}^{*}$ are the baseline magnetic relaxation rates without contrast agent $\left(R_{2,0}^{*}\right)$ for extravascular and intravascular space, respectively.

As shown in Eq. 1, separate signal exponentials are added together for each vascular compartment within a voxel for *S*_*IVS*_, however, relaxation rates are added from each vascular compartment within **a single** exponential for *S*_*EES*_, as will be described in Eq. 5. As a reminder, it is intravascularly contained contrast agent that gives rise to ${\text{T}}_{2}^{*}$ signal change in both the intravascular and extravascular spaces (please see the Discussion section for leakage of contrast agent into the extravascular space).

#### Extravascular and intravascular relaxation rates

At 3T, $R_{2,0,EES}^{*}$ and $R_{2,0,IVS}^{*}$ have been experimentally approximated to be 20.99 s^−1^ and 13.8 s^−1^ for fully oxygenated blood, respectively (Zhao et al., 2007; Uludag et al., 2009). Polynomial equations that model $R_{2,con,EES}^{*}$ for various vessel radii have been determined using Monte-Carlo simulations; for a large range of susceptibility values at 3T, the equations (expressed as per one percent of *CBV*) can be simplified by a linear relationship (see Table 1 and Uludag et al., 2009):


(4) $$\begin{array}{l} \Delta v_{dOHb}=0.264\cdot Hct\cdot \left(1-Y_{0}-{\Delta Y}_{dOHb}\right)\cdot \gamma {\cdot B}_{0} \end{array}$$

**Table 1:** Coefficients for extravascular relaxation rates in various vessel radii.

**Vessel radius**	**Relaxation equation**
5 μm	$R_{2,con,EES,i}^{*}=\;$0.0387·Δ*v*
16 − 200 μ*m*	$R_{2,con,EES,i}^{*}=\;$0.0433·Δ*v*
200 μ*m*, 90^*o*^	$R_{2,con,EES,i}^{*}=\;$0.0798·Δ*v*

5 μm (capillary), 16 − 200 μ*m* (large artery and vein randomly oriented to the magnetic field, arteriole, and venule), and 200 μ*m*, 90^*o*^ (artery and vein perpendicular to the magnetic field) are the simulated vessel radii. Note that the large artery and vein can also be chosen to be randomly oriented, as is shown in Uludag et al., 2009, which affects the quantitative predictions but not the main findings (data not shown). Δ*v* represents the frequency shift at the surface of the vessel in the presence of a contrast agent, shown in Eq. 4 and Eq. 7.

Δ*v*_*dOHb*_ is the frequency shift induced by dOHb, *Hct* is the hematocrit fraction (simulated as 0.4), Δ*Y*_*dOHb*_ is the percentage change in the oxygenation as a decimal (which corresponds to changes in dOHb), γ is the gyromagnetic ratio (2π·42.6 *MHz*/*T*), and *B*_0_ is the magnetic field strength (3T). *Y*_0_ represents the baseline oxygen saturation as a decimal, dependent on the vascular compartment (refer to Table 2). $R_{2,con,EES}^{*}$ can then be determined by summating $R_{2,con,EES,i}^{*}$ for each vessel compartment within the voxel:


(5) $$\begin{array}{l} R_{2,con,EES}^{*}=\sum_{i}(pCBV_{i}\cdot R_{2,con,EES,i}^{*}) \end{array}$$

**Table 2:** Summary of simulated voxel properties for arterial, tissue, and venous voxels.

	**Arterial**	**Tissue**	**Venous**
***CBV*** **(%)**	10–100	4	10–100
***MTT*** **(s)**	N.A.	3	4
**Delay (s)**	0	1	2
**Vessel Composition**	90° Artery	Artery / Arteriole; Capillary; Vein / Venule (1:2:2)	90° Vein
**Y**_**0**_ **(%)**	100	Artery (100); Arteriole (95); Capillary (77.5); Vein/Venule (60)	60

The values in this table can be used for any arbitrary set of physiological assumptions [i.e., here tissue represents gray matter (GM), but *CBV* can be changed to 2% to simulate white matter (WM) as well]. Vessel ratios and Y_0_ for each vessel type were informed by previous work (Vovenko, 1999; Lu and Ge, 2008; Uludag et al., 2009). The arterial voxel is comprised of extravascular space and an artery that is oriented 90° to the main magnetic field, where the fraction of intravascular space is scalable from 0 to 100% (Eq. 1). Similarly, the venous voxel is comprised of extravascular space and a vein that is oriented 90° to the main magnetic field. These large vessels were simulated as perpendicular to the main magnetic field to mimic the stem of the middle cerebral artery (MCA) and dorsal portion of the superior sagittal sinus (SSS), which are both roughly perpendicular to the main magnetic field. *MTT* and delay were based on previous findings (Ibaraki et al., 2007). Once these parameters were selected, within the range denoted in the table, they were incorporated into the signal model as described in Supplementary Figure 3.

Here, *i* denotes an individual vessel compartment and *pCBV*_i_ represents the percentage blood volume of the vessel contributing to the signal. Note that *pCBV*_i_ must be a percentage and not a decimal (i.e., for a blood volume of 5%, a value of 5 is used as opposed to 0.05), as the equations for $R_{2,con,EES,i}^{*}$ in Table 1 are modeled for one percent of *CBV*.

Finally, the $R_{2,con,IVS}^{*}$ dependence on dOHb at 3T (*Hct* ≈ 0.4) was found to be quadratic from previous blood phantom work (Zhao et al., 2007):


(6) $$\begin{array}{l} R_{2,con,IVS}^{*}=181\cdot {\left(1-Y_{0}-{\Delta Y}_{dOHb}\right)}^{2} \end{array}$$

#### Quantifying the relationship between dOHb and Gd

The above equations quantify MRI signal in response to changes in dOHb. To calculate the signal change induced by Gd, the effect of Gd on $R_{2,con,IVS}^{*}$ and $R_{2,con,EES}^{*}$ must be determined. One way to determine this is by finding a quantitative relationship between Gd and dOHb; given that dOHb and Gd are both paramagnetic contrast agents, a certain increase in [dOHb] will induce the same susceptibility effect as 1 mM of Gd.

The frequency shifts on the surface of the blood vessel caused by changes in dOHb and Gd are known. The frequency shift induced by dOHb is described in Eq. 4 while the frequency shift induced by Gd (Δ*v*_*Gd*_), when *Hct* ≈ 0.4, is described by the following equation, derived from Kjølby et al. (2006):


(7) $$\begin{array}{l} \Delta v_{Gd}=\gamma {\cdot B}_{0}\cdot 0.026\cdot \left[Gd\right] \end{array}$$

[*Gd*] represents the concentration of Gd (mM) in blood, and 0.026 ppm/mM represents the molar susceptibility of Gd, specifically Gd-DTPA (van Osch et al., 2003). Setting Eq. 7 equal to Eq. 4 allows for the interconversion of percentage dOHb saturation and Gd concentration (mM). This interconversion is an important step in being able to simulate and compare dOHb- and Gd-induced signal changes with the same DSC MRI framework:


(8.1) $$\begin{array}{l} 0.264\cdot 0.4\cdot \left({1-Y_{0}-\Delta Y}_{dOHb}\right)\cdot \gamma {\cdot B}_{0}=\gamma {\cdot B}_{0}\cdot 0.026\cdot \left[Gd\right]\\ \;+\;0.264\cdot 0.4\cdot \left(1-Y_{0}\right)\cdot \gamma {\cdot B}_{0} \end{array}$$

(8.2) $$\begin{array}{l} \;Y_{dOHb}=-0.246\cdot \left[Gd\right] \end{array}$$

By setting Δ*v*_*dOHb*_ equal to *v*_*Gd*_ at 3T, a certain concentration of Gd associated with magnetic susceptibility can be converted to a value of blood oxygenation and vice versa. For example, a 1 mM increase in Gd is equivalent in susceptibility to a 24.6% increase in dOHb at 3T. This equivalency is referred to as the pseudo-oxygenation (the oxygenation change that has the same susceptibility effect as 1 mM of Gd on the magnetic field at 3T). Eqs. 1–6 were then used to simulate either a Gd- or dOHb-induced bolus by substituting Δ*Y*_*dOHb*_ with −0.246·[*Gd*] or using Δ*Y*_*dOHb*_, respectively. Thus, it is important to note that Gd and dOHb were modeled with the same signal framework, with differences only in the input bolus properties (i.e., susceptibility, concentration, shape, and duration) and compartmentalization in the vasculature. This idea can be further understood in Supplementary Figure 1.

### Section 2: Model inversion

#### Relaxation curves

The first step in standard DSC analysis, following preprocessing, is the conversion of a signal time course (*S(t)*) into a relaxation rate time course ($\Delta R_{2}^{*}\left(t\right))$:


(9) $$\begin{array}{l} \Delta R_{2}^{*}\left(t\right)=-\left(\frac{1}{TE}\right)\cdot \ln\left(\frac{S\left(t\right)}{S_{0}}\right) \end{array}$$

This step requires the specification of a baseline signal (*S*_0_), along with the *TE*. Of note, as intravascular relaxation is quadratically related to contrast agent, some studies have opted for a quadratic equation as opposed to Eq. 9 to solve the concentration time course in artery or vein (see references in Calamante, 2013). While we tested this method, we do not apply it here as it assumes that the selected arterial voxel is entirely intravascular, and due to partial volume errors, this is almost never the case.

#### Calculations of cerebral blood volume, cerebral blood flow, and mean transit time

From this point forward, the term “relative” will be used to describe tissue signal normalized by signal from a reference voxel (arterial or venous). Once tissue, arterial, and venous signal time courses are converted into $\Delta R_{2}^{*}$(t), the relative *CBV* (*rCBV*) can be calculated (Knutsson et al., 2010). Of note, normalizing tissue to venous as opposed to arterial signal is of clinical interest, as venous voxels tend to have reduced partial volume effects (Calamante, 2013). However, we only normalize to venous signal for the purposes of calculating *rCBV*, as doing so for *CBF* and *MTT* would violate principles of indicator dilution theory in our method (Meier and Zierler, 1954); this is because we normalize tissue time courses directly to the venous time courses, and do not first scale the arterial time course by the area under the venous curve. Thus, performing a deconvolution of tissue data from the venous time course would not accurately reflect *CBF* and *MTT*.

*rCBV* is typically scaled by κ, the hematocrit correction factor [(1−*Hct*)/(1 − 0.69*Hct*)] when using Gd contrast (Tudorica et al., 2002). However, when using dOHb contrast, we concluded that the κ value should be set to *Hct*/0.69*Hct*, as contrast is limited to the red blood cell fraction and not the plasma.

The relative *CBF* (*rCBF*) and *MTT* are calculated based on indicator dilution theory and the central volume principle, respectively (Meier and Zierler, 1954). These values were estimated using discretized singular value decomposition (Østergaard et al., 1996; Wirestam et al., 2000) with a noise threshold of 20%, which was determined to be most effective in previous work (Bjørnerud and Emblem, 2010).

The simulation framework and analysis pipeline are visually summarized in Supplementary Figure 2.

## Methods

### Part 1: Simulation methods

#### Arterial, tissue, and venous concentration input functions

The forward model, as described above, can be used to model any shape, duration, or concentration of Gd and dOHb input time courses. For the examples in this study, we generally assume parameters closely resembling experimental data.

The Gd input time course [Gd(t)] in an artery is well-modeled as a gamma variate function (Thompson et al., 1964; Davenport, 1983).


(10) $$\begin{array}{l} Gd\left(t\right)=a\cdot {\left(\frac{t}{b}\right)}^{c}\cdot e^{\left(c\cdot \left(1-\frac{t}{b}\right)\right)} \end{array}$$

While it is difficult to attain the exact concentration profile of Gd in an artery (Patil et al., 2013; Kellner et al., 2018; Lind et al., 2020), the shape parameters, *b* (time to peak) and *c*, are set to 2.5 s and 3 (unitless), respectively, to model the shape observed experimentally. *a* (the peak concentration) is set to approximately 2 mM—see Discussion. The dOHb input, as informed by our experimental validations and previous work (Poublanc et al., 2021), was modeled as a rectangular hypoxic bolus of roughly 30 s duration, convolved with a decaying mono-exponential as the bolus takes time to reach the target oxygenation and enter the cerebral vasculature.

To attain the tissue concentration profile, the arterial input was convolved with a well-defined biexponential residue function [*R(t)*] which designates fast and slow flowing vascular compartments (Mehndiratta et al., 2014):


(11.1) $$\begin{array}{l} R\left(t\right)=f\cdot e^{-t\cdot t1}+\left(1-f\right)\cdot e^{-t\cdot t2} \end{array}$$

Here *t1* (fast-flowing compartment transit time), *t2* (slow-flowing compartment transit time), and *f* (fraction of flow in the fast-flowing compartment), are vascular flow parameters which yield the *MTT* in the following equation (Mehndiratta et al., 2014):


(11.2) $$\begin{array}{l} MTT=\frac{f}{t1}+\frac{1-f}{t2} \end{array}$$

As we expect the venous voxel to be generalizable to a single vascular compartment, we convolve the arterial input with a mono-exponential residue function to obtain the venous concentration profile (Mehndiratta et al., 2014):


(12) $$\begin{array}{l} R\left(t\right)=e^{-\frac{t}{MTT}} \end{array}$$

The flow parameters, informed by clinical data and literature yield a tissue voxel *MTT* of roughly 3 s (*f* = 0.92, *t*_1_ = 0.68, *t*_2_ = 0.05) and a venous voxel *MTT* of 4 s (*MTT* = 4) (Ibaraki et al., 2007). As well, a delay of 1 s and 2 s were applied to the tissue and venous voxels, respectively (Ibaraki et al., 2007). These parameters are easily adjustable to model other values.

#### Simulated voxel parameters

The voxel parameters used for the simulations were based off previously reported physiological estimates and are summarized in Table 2.

### Part 2: Experimental methods

#### Subjects

This study was approved by the Research Ethics Board of the University Health Network, Toronto, Canada, and conforms to the Declaration of Helsinki. Written informed consent was obtained in all 6 healthy volunteers (age range 22–60, 1 woman). All subjects were non-smokers and not taking regular medication. While our sample size is rather small, the results were consistent across all subjects.

#### Scan protocols and MRI sequences

The RespirAct™ RA-MR (Thornhill Medical, Toronto, Canada) was used to target lung S_a_O_2_ while maintaining isocapnia (Sobczyk et al., 2016). The maintenance of isocapnia ensures that vasodilation and *CBF* changes are mitigated. All gases and sensors were calibrated prior to use. GRE-EPI and anatomical T_1_ scans were acquired on a 3T MRI system (Signa HDx—GE Healthcare, Milwaukee) with echo-planar (EPI) acquisition.

MR parameters for dOHb and Gd GRE scans were TR/TE = 1,500/30 ms, flip angle = 73°, 3 mm isotropic voxels, 29 contiguous slices—no interslice gap. The coverage is lower as the scanner does not have multi-band capabilities, although the coverage is not a relevant parameter for our results. The dOHb scan duration was approximately 210 s per paradigm; the Gd scan duration was approximately 90 s. A high-resolution T_1_-weighted 3D spoiled GRE sequence was acquired for each subject as well (TI = 450 ms, TR = 7.88 ms, TE = 3 ms, flip angle = 12°, voxel size = 0.859 x 0.859 x 1 mm, matrix size = 256 x 256, 146 slices, field of view = 24 x 24 cm, no interslice gap).

**Figure 1:**
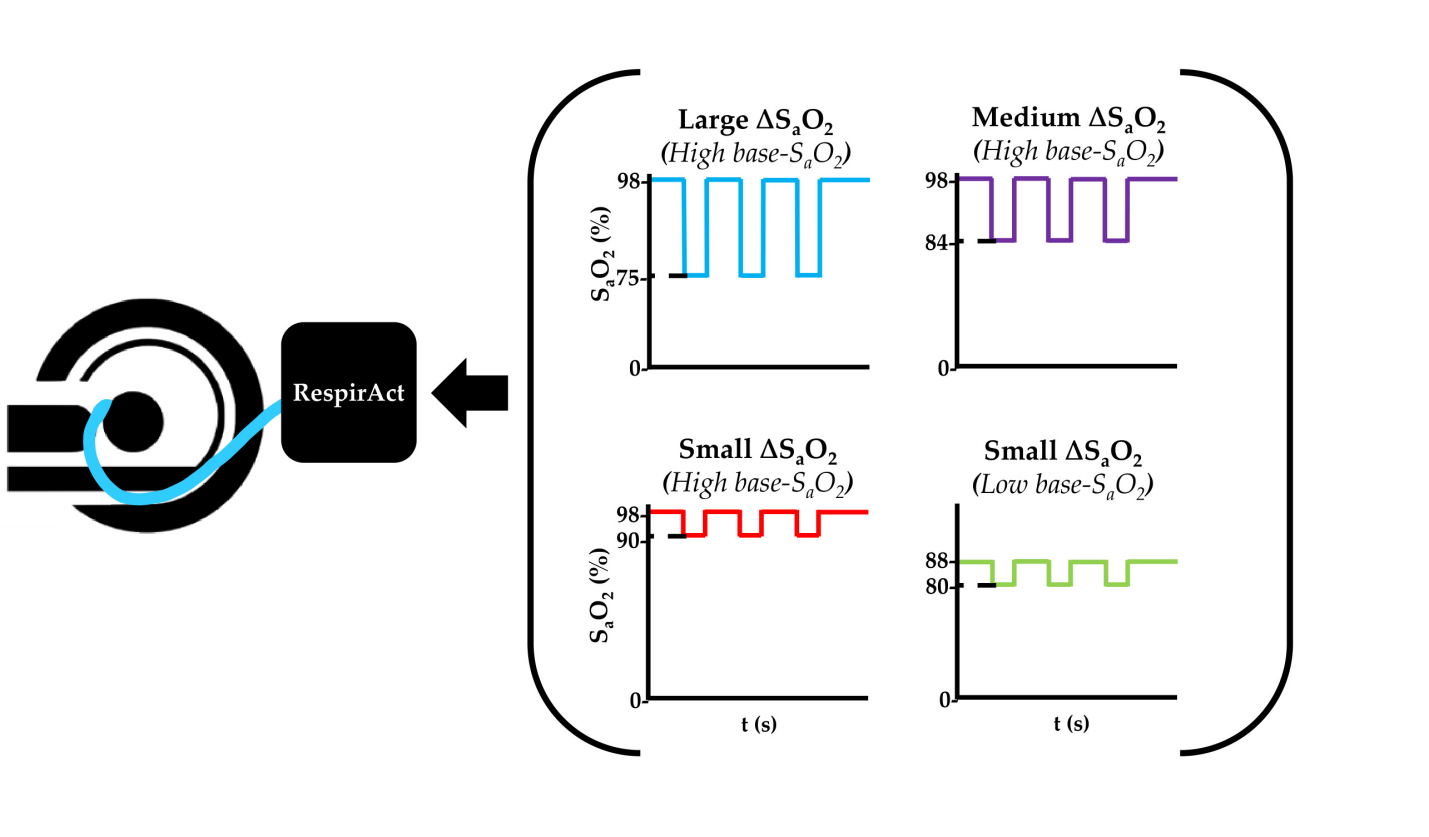
Summary of the dOHb Experimental Method. Subjects (*n* = 6) were imaged in a 3T MRI scanner with a mask attached to the RespirAct^®^, where S_a_O_2_ was altered to attain desired levels of base-S_a_O_2_ and ΔS_a_O_2_. In sum, the following paradigms were generated: small ΔS_a_O_2_ with high base-S_a_O_2_ (red: 98–90% S_a_O_2_), medium ΔS_a_O_2_ with high base-S_a_O_2_ (purple: 98–84% S_a_O_2_), large ΔS_a_O_2_ with high base-S_a_O_2_ (blue: 98–75% S_a_O_2_), and small ΔS_a_O_2_ with low base-S_a_O_2_ (green: 88–80% S_a_O_2_).

In the dOHb experiments, the hypoxic bolus magnitude (ΔS_a_O_2_) and/or baseline oxygen saturation (base-S_a_O_2_) were varied (Figure 1). To determine the effect of ΔS_a_O_2_ and base-S_a_O_2_ for perfusion quantification, S_a_O_2_ values were modified to generate four hypoxic paradigms with three ~30 s hypoxic boluses as follows: 98–90%, 98–84%, 98–75%, and 88–80%. Each hypoxic drop was acquired over multiple breaths, depending on the rate and volume of ventilation, as attaining the hypoxic state required wash-out of oxygen from the functional residual capacity of the lungs (air remaining after exhalation). Due to this physiological limitation, the dOHb bolus is longer in duration that the Gd bolus, which depends on the speed of injection and dispersion of the bolus in its trajectory from peripheral vein to the brain. The hypoxic target was maintained for approximately 30 s before returning to baseline in a single breath. The rapidity of the return to baseline was due to the higher available oxygen partial pressure target (713 mmHg) relative to the hypoxic target (<100 mmHg). The baseline before and after each bolus was 30 s in duration.

The dOHb paradigms were acquired prior to the Gd paradigm such that Gd would not remain in the system as a steady-state and influence the dOHb data. Here, 5 mL of 1 M gadobutrol (Gadovist^®^) was injected at 5 mL/s followed by 30 mL of saline at 5 mL/s. This corresponds to a dose that is slightly below the standard dose of 0.1 mmol/kg for the average subject.

One subject reported a mild change in respiratory sensation during the dOHb protocol. Two subjects reported discomfort and slight nausea during Gd acquisitions. Only data from one paradigm of one subject was removed due to excessive motion during the scan.

#### Preprocessing

FSL (version 6.0.4) was used for image preprocessing (Woolrich et al., 2009). Brain extraction was performed on T_1_ anatomical images; brain extraction, motion correction, and interleaved slice time correction were performed on dOHb and Gd scans. Gaussian temporal filtering was only performed on the dOHb scan by averaging each signal time point by a 1 × 5 Gaussian kernel; doing so for the Gd bolus was not required due to high signal-to-noise. For all dynamic scans, the first 2–3 timepoints were removed to allow for signal equilibration. Percentage maximal signal change (Δ*S*) and the signal to noise ratio (*SNR*) were calculated:


(13) $$\begin{array}{l} \Delta S=\frac{\left(S_{0}-S_{Peak}\right)}{S_{0}}\cdot 100 \end{array}$$

(14) $$\begin{array}{l} SNR=\frac{\Delta S}{\varepsilon_{t}\;} \end{array}$$

In Eq. 13, *S*_0_ is the average signal of ten temporal volumes before and after the selected bolus, and *S*_*Peak*_ is the maximum signal drop. The *SNR* is computed as shown in Eq. 14, where ε_*t*_ is the standard deviation of signal for ten temporal volumes before and after the selected bolus divided by *S*_0_. *S(t)* was then converted to $\Delta R_{2\;}^{*}\left(t\right)$ using Eq. 9. The above equations were applied to dOHb and Gd signal data from all subjects using Python 3.7.6.

#### Selection of arterial and venous voxels

Typically, in a DSC MRI analysis, the ${\text{T}}_{2}^{*}$-weighted DSC data is registered to high-resolution T_1_-weighted anatomy. In this study, to reduce registration errors and the misidentification of tissue classes, we sought to identify arterial and venous voxels in the functional rather than anatomical space. However, the main results are not affected by either analysis choice. As arterial voxels are difficult to clearly identify on functional images, we used the following conditions for voxel selection: Arterial voxels were defined based on their proximity to the M1 segment of the middle cerebral artery (MCA) using both functional and registered T_1_ anatomical data, Δ*S* within the top 10% of the Δ*S* values, and short delay (~0–1 s). Venous voxels were defined based on their proximity to the superior sagittal sinus (SSS) using registered T_1_ anatomical data, ΔS within the top 10% of the ΔS values, long delay on the Gd and dOHb maps (~2 s), and low *S*_0_ (as vein contains more dOHb than artery, reducing the baseline signal). Once the voxels were selected on the Gd map, they were linearly registered to the dOHb paradigms to avoid displacement due to motion between the runs.

We recognize that for the purposes of maintaining better inter-subject agreement, it is often better to take an average of arterial or venous candidate voxels. However, for the questions that we were trying to address in our study, we wanted to ensure that the voxel selected was mostly intravascular, and thus sought to avoid averaging between multiple candidate voxels by selecting one voxel.

#### Perfusion quantification

After defining an arterial input function (AIF) from the M1 segment of the MCA or venous output function (VOF) from the dorsal section of the SSS, each time series was truncated to begin at the start of the AIF bolus and finish at the end of the VOF bolus. Then, *rCBV, rCBF*, and *MTT* for each voxel were calculated (see Theory section, above). Any voxel yielding a negative *rCBV* was excluded, observed in voxels with high noise and low blood volume (roughly 10% of the voxels for Gd and 20% of the voxels for dOHb).

#### Gray and white matter segmentation

As mentioned above, while DSC MRI studies typically employ T_1_ anatomical segmentation to DSC maps due to higher anatomical contrast in T_1_ images relative to baseline ${\text{T}}_{2}^{*}$ images, this form of segmentation requires image registration. In the current study, we use a segmentation method in the native space to avoid registration errors: GM exhibits larger signal changes in ${\text{T}}_{2}^{*}$ images than WM and can therefore be easily distinguished. Nevertheless, applying these different segmentation strategies does not affect the main findings of this study. The *ΔS* functional maps for Gd were segmented into gray matter (GM) and white matter (WM) using a threshold method. WM voxels were defined as those containing Δ*S* values within the lowest 15–20% of the Δ*S*_*Gd*_ data range, while GM was defined as voxels containing the highest 75% *ΔS* values. The maps were then binarized and linearly registered to the functional space of each dOHb paradigm. The GM and WM regions were further masked with a thresholded ε_*t*_ map (voxels with values in the lowest 10% of the ε_*t*_ range were kept in the mask) to remove noisy voxels in peripheral brain tissue and skull base. The exclusion of voxels based on ε_*t*_ also removes large arterial and venous voxels from the GM masks due to high levels of physiological noise in these regions. The GM and WM were finally masked with a thresholded Gd *MTT* map (voxels within the lowest 70% of robust range of *MTT* were kept in the mask) to remove voxels in the CSF (Supplementary Figure 4).

#### Statistical testing

Statistical testing was conducted for some of the experimental data by applying a two-tailed student's *t*-test, with an alpha level of 0.05. As we only compare mean values of various parameters with simulation-guided hypotheses, we believe this statistical method is sufficient. Linear regressions were also conducted for voxel-wise comparisons.

#### Data and code availability

Anonymized data will be shared by request from any qualified investigator for purposes such as replicating procedures and results presented in the article, so long as the data transfer agrees with the University Health Network and Health Canada legislation on the general data protection regulation.

Code used for the simulations can be found on GitHub through the following link: https://github.com/jschulman-1998/NeuroImage2022.

## Results

**Figure 2:**
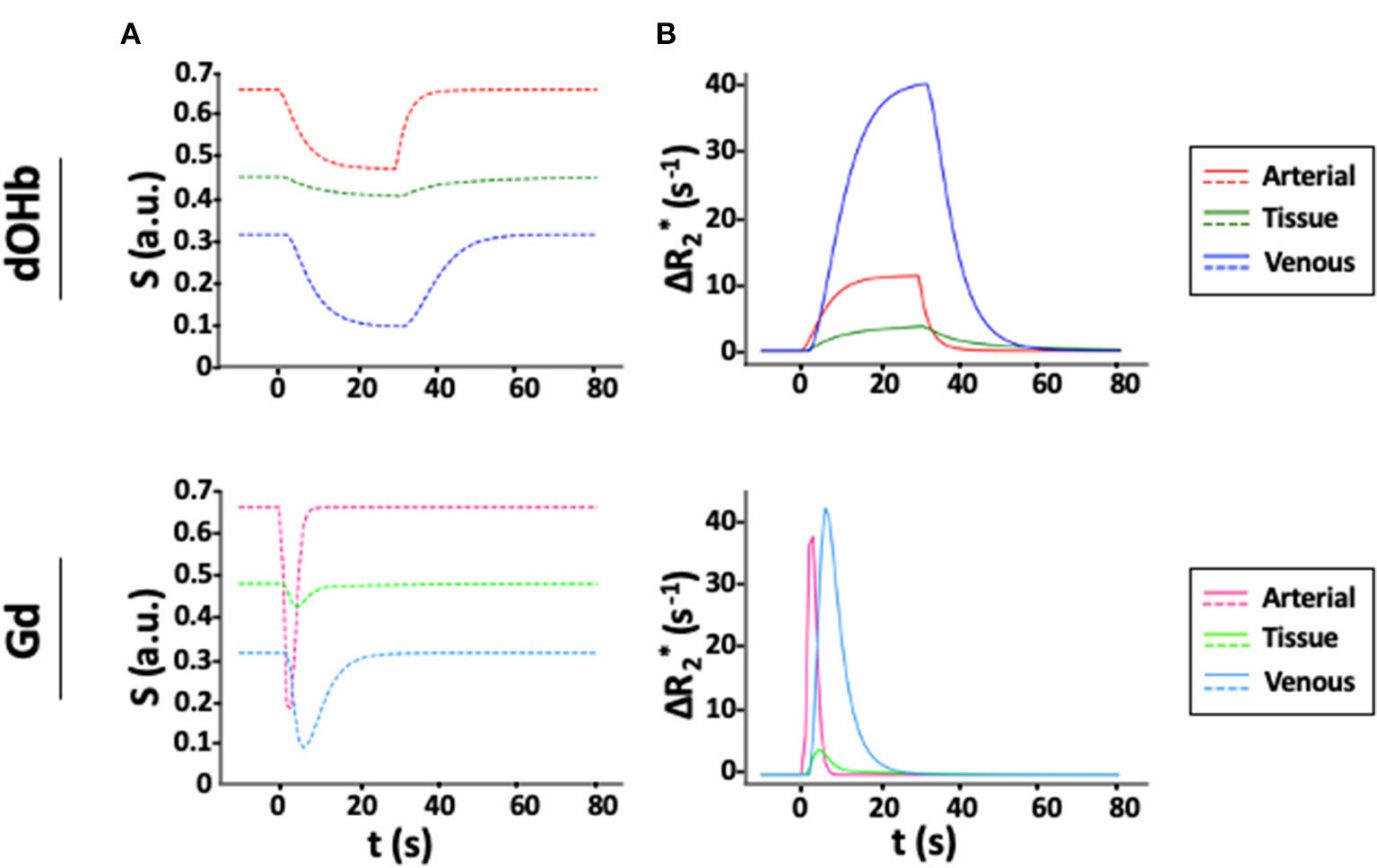
Simulated *S(t)* and $R_{2}^{*}\left(t\right)$ for Gd and dOHb Contrast. **(A)** *S(t)* in artery (*CBV* = 100%), tissue (*CBV* = 4%, *MTT* = 3 s), and vein (*CBV* = 100%, *MTT* = 4 s) following contrast agent administration of dOHb (top) and Gd (bottom). **(B)** Corresponding $R_{2}^{*}\left(t\right)$. Note that the simulated dOHb profile follows a 98–75% S_a_O_2_ and Gd profile has a peak input concentration of 2 mM.

Figure 2 displays *S(t)* and $\Delta R_{2\;}^{*}\left(t\right)\;$for simulated tissue, arterial, and venous voxels in response to a bolus of either Gd or dOHb. The baseline signal is highest for artery and lowest for vein due to differences in baseline oxygen saturation. In addition, the dOHb boluses are longer than Gd boluses, mimicking experimentally observed bolus durations (roughly 30–40 s for hypoxic boluses). According to this simulation, $\Delta R_{2,\max}^{*}$, the peak of $\Delta R_{2\;}^{*}\left(t\right)$, attained with dOHb is roughly 4 times larger in vein relative to artery even though the same ΔS_a_O_2_ is simulated for artery and vein, indicating a substantial effect of the baseline oxygen saturation on $\Delta R_{2\;}^{*}\left(t\right)$. Remarkably, for Gd, $\Delta R_{2,\max}^{*}$ is only roughly 1.1 times larger in vein relative to artery, indicating a reduced effect of the baseline oxygen saturation on $\Delta R_{2\;}^{*}\left(t\right)$ for contrast agents with a higher susceptibility. As well, the ratio of arterial to tissue $\Delta R_{2,\max}^{*}$ is much higher when using Gd in comparison with dOHb. Conversely, the ratio of venous to tissue $R_{2,\max}^{*}$ is much more consistent between Gd and dOHb.

**Figure 3:**
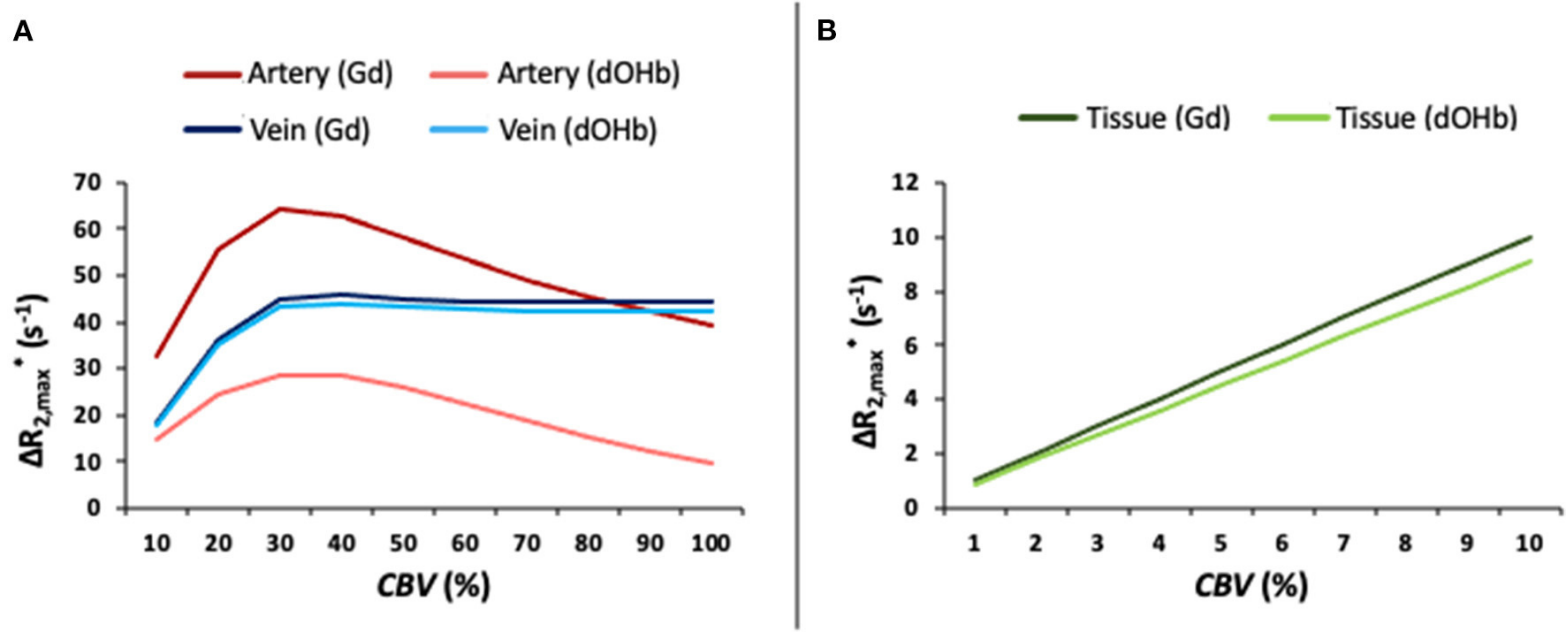
$\Delta R_{2,\max}^{*}\;$as a Function of Simulated *CBV* (%). **(A)** $\Delta R_{2,\max}^{*}\;$as a function of the simulated *CBV* for artery and vein in response to Gd and dOHb. **(B)** $\Delta R_{2,\max}^{*}\;$as a function of the simulated *CBV* for tissue (*CBV*_*WM*_ ≈ 2%; *CBV*_*GM*_ ≈ 4%) in response to Gd and dOHb. Note that the simulated dOHb bolus follows a 98–75% S_a_O_2_ and Gd bolus has a peak input concentration of 2 mM.

Figure 3 provides an analysis of the maximal relaxation rate ($\Delta R_{2,\max}^{*})$ in artery, tissue, and vein as a function of the simulated *CBV*, mimicking partial volume effects. For tissue, linear changes in the simulated *CBV* result in linear increases in the $\Delta R_{2,\max}^{*}$, in response to either contrast agent. This is not the case for arterial and venous responses. For the arterial response to dOHb, increasing *CBV* increases the $\Delta R_{2,\max}^{*}$ up to a certain *CBV* (roughly 30%), such that any further increase in *CBV* leads to decreases in $\Delta R_{2,\max}^{*}$. The simulated arterial *CBV* to $\Delta R_{2,\max}^{*}$ relationship in response to Gd also has a maximum for an intermediate *CBV* value. For venous voxels, $\Delta R_{2,\max}^{*}$ saturates at high *CBV* values in response to either Gd or dOHb. These results point to a complex relationship between relaxation rate changes and voxel composition, which is further elaborated upon in Supplementary Figure 5.

**Figure 4:**
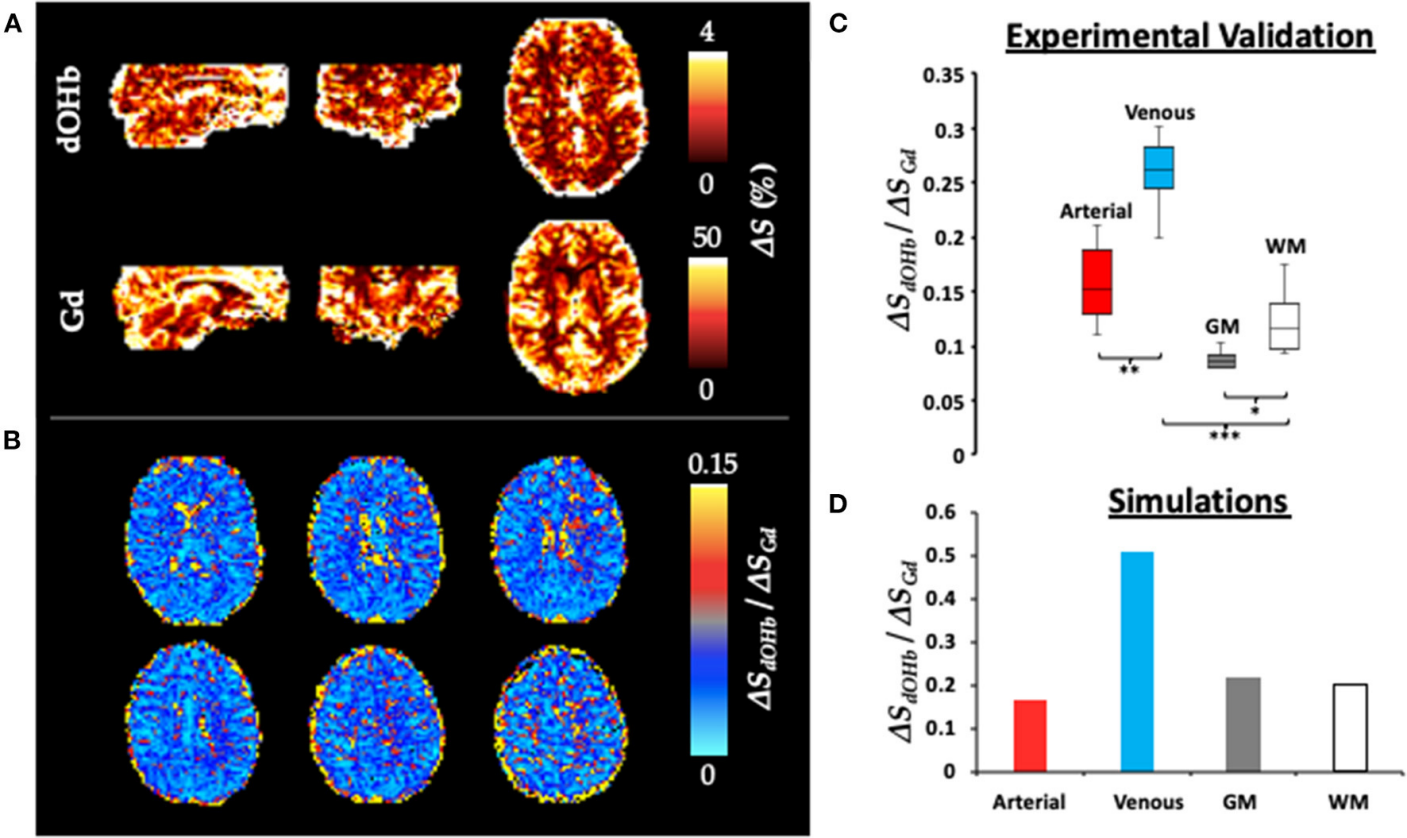
Δ*S* for Gd and dOHb in Artery, Vein, GM, and WM. **(A)** Δ*S* (%) maps (representing Δ*S*_*max*_) for Gd and dOHb in a representative subject. Note that the dOHb map here is an average of the following paradigms: small ΔS_a_O_2_ with high base-S_a_O_2_, medium ΔS_a_O_2_ with high base-S_a_O_2_, large ΔS_a_O_2_ with high base-S_a_O_2_. **(B)** Six representative axial slices of Δ*S*_*dOHb*_ map divided by Δ*S*_*Gd*_ map. **(C)** Box and whisker plot (average from all subjects) of Δ*S*_*dOHb*_*/*Δ*S*_*Gd*_ for various tissue compartments, including artery (red), vein (blue), GM (gray), and WM (white). Significant differences are denoted with asterisks. **(D)** Δ*S*_*dOHb*_*/*Δ*S*_*Gd*_ for simulated artery (red), vein (blue), GM (gray), and WM (white) voxels. Simulated dOHb bolus followed a 98–84% S_a_O_2_ profile. Simulated Gd bolus has a peak concentration of 6 mM. Simulated *CBV* values were as follows: artery (90%), vein (90%), GM (4%), and WM (2%).

Figure 4 shows Δ*S* maps, simulation data, and collective experimental results in response to dOHb and Gd boluses. For simplicity here and elsewhere, Δ*S* represents Δ*S*_*max*_ and is displayed as an absolute signal change. Δ*S*_*Gd*_ is ~10x higher than Δ*S*_*dOHb*_. When the Δ*S*_*dOHb*_ values are divided by the Δ*S*_*Gd*_ values in corresponding voxels, hereafter termed the Δ*S* ratio, differences between artery, vein, CSF, GM, and WM are visually highlighted (see Supplementary Figure 4 for masks of these regions). The Δ*S* ratio is significantly different in each vascular/tissue compartment, as evident from the box and whisker plot in Figure 4, except for artery vs. WM. The experimental finding of the Δ*S* ratio being significantly higher in venous voxels in comparison to tissue voxels (GM and WM) is corroborated by simulation findings, which show that the venous Δ*S* ratio is larger than either the GM or WM Δ*S* ratio. The experimental finding of the Δ*S* ratio being higher in arterial voxels in comparison to tissue voxels (GM and WM) is not corroborated by simulation findings when the artery is simulated at a *CBV* of 90%, as is shown here, but is when artery is simulated at a lower blood volume. The WM Δ*S* ratio being higher than the GM Δ*S* ratio, although present in the experimental data, is not observed in the simulations—refer to Discussion.

**Figure 5:**
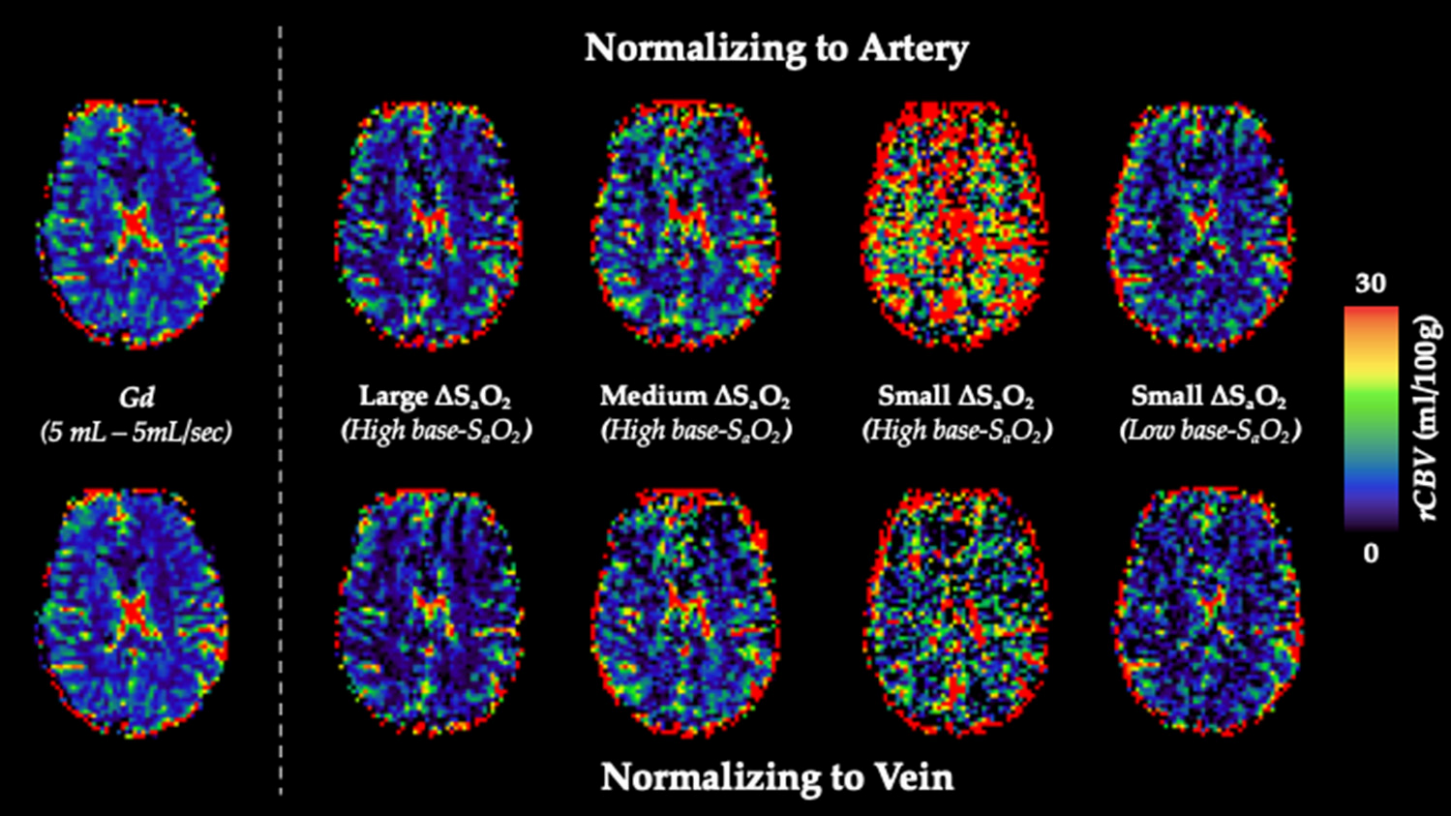
*rCBV* Dependency on ΔS_a_O_2_ and Base-S_a_O_2_. **Top (Arterial Normalization)**. *rCBV* (mL/100 g) maps from representative subject axial slice where tissue is normalized to an arterial voxel in the MCA for five subsequent paradigms (left to right): standard Gd, large ΔS_a_O_2_ with high base-S_a_O_2_, medium ΔS_a_O_2_ with high base-S_a_O_2_, small ΔS_a_O_2_ with high base-S_a_O_2_, small ΔS_a_O_2_ with low base-S_a_O_2_. **Bottom (Venous Normalization)**. *rCBV* (mL/100g) maps from representative subject axial slice where tissue is normalized to a venous voxel in the SSS for the same paradigms.

**Figure 6:**
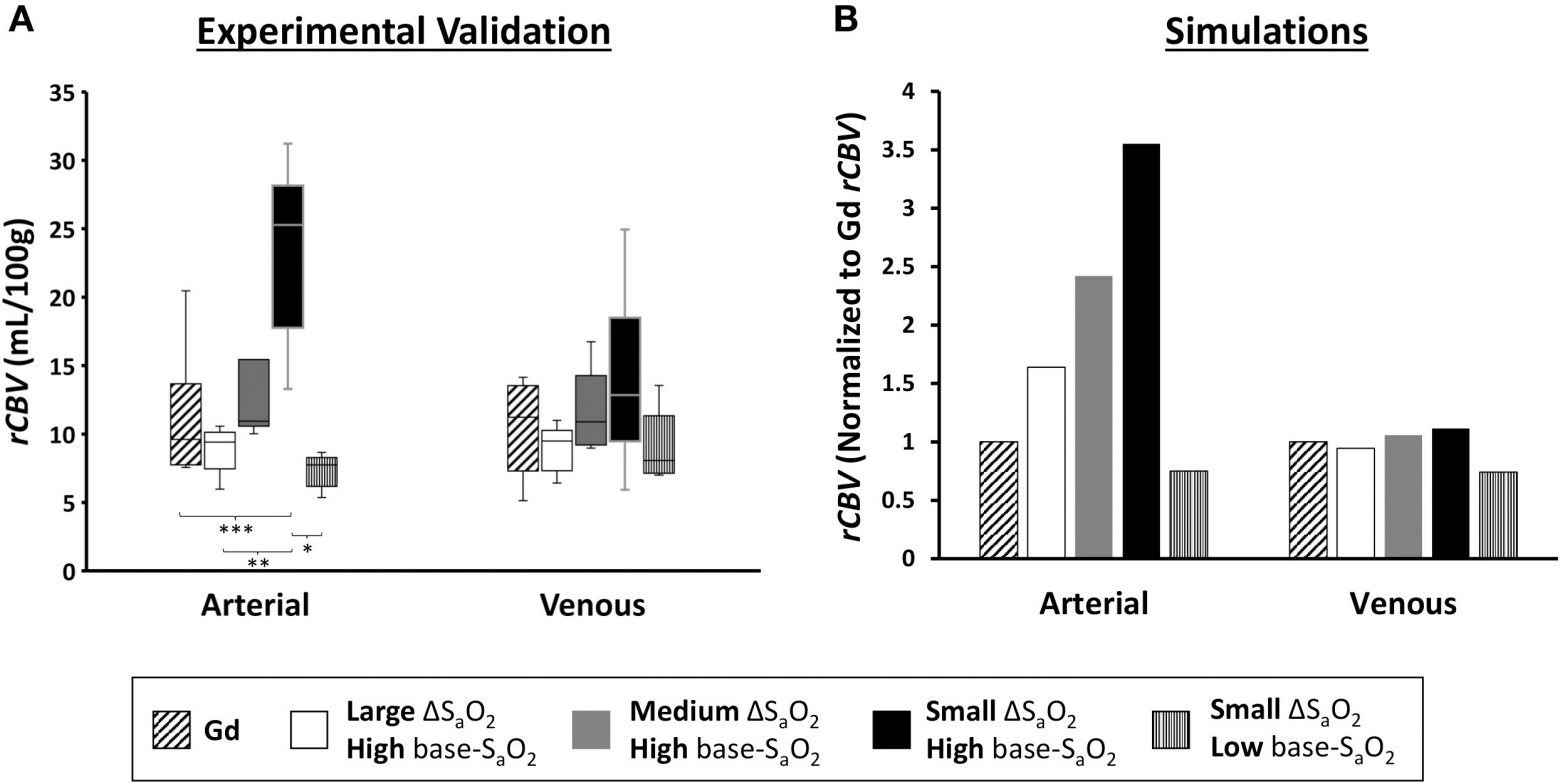
*rCBV* Dependency on Arterial vs. Venous Normalization. **(A)** Validations. Box and whisker plot of calculated *rCBV* when normalizing tissue to arterial voxel in the MCA vs. venous voxel in the SSS (*n* = 6) for Gd and dOHb at varying ΔS_a_O_2_ and Base-S_a_O_2_. Significant differences are denoted with asterisks. **(B)** Simulations. Calculated *rCBV* when normalizing tissue to simulated artery or vein for the same paradigms as in the experiments. Calculated values for all paradigms are further normalized to the *rCBV* attained with Gd (values above one are an overestimation relative to Gd). Simulated tissue *CBV* = 4%; Simulated arterial *CBV* = 100%; Simulated venous *CBV* = 100%.

**Figure 7:**
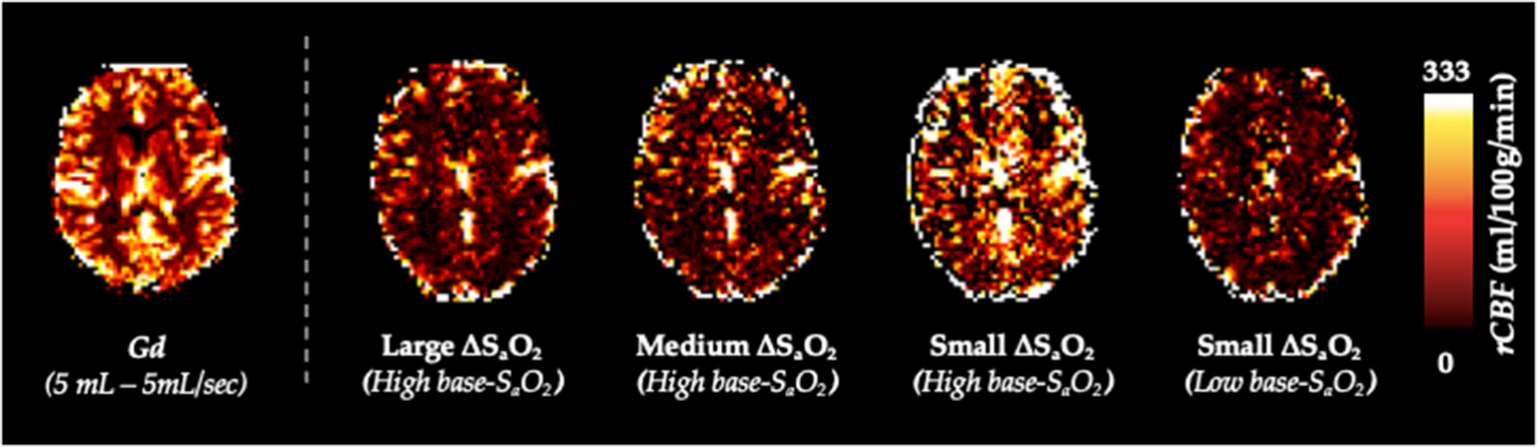
*rCBF* Dependency on ΔS_a_O_2_ and Base-S_a_O_2_. *rCBF* (mL/100g/min) maps from representative subject axial slice where tissue is normalized to an arterial voxel in the MCA for five subsequent paradigms (left to right): standard Gd, large ΔS_a_O_2_ with high base-S_a_O_2_, medium ΔS_a_O_2_ with high base-S_a_O_2_, small ΔS_a_O_2_ with high base-S_a_O_2_, small ΔS_a_O_2_ with low base-S_a_O_2_.

Figure 5 shows calculated *rCBV* maps from the Gd and dOHb paradigms in a representative subject when normalizing the tissue relaxation rate time courses to that of a selected arterial (top) or venous voxel (bottom). This is elaborated upon in Figure 6, where cumulative *rCBV* results from all subjects in addition to simulation results are presented for various paradigms. Both figures show that the calculated *rCBV* depends on the molar susceptibility of contrast agent (Gd vs. dOHb), amount of contrast agent applied, baseline susceptibility (base-S_a_O_2_), and choice of normalizing voxel (artery or vein). In the experimental validations and simulations, the order of *rCBV* estimation for the dOHb paradigms, from lowest to highest, is as follows: small ΔS_a_O_2_ with high base-S_a_O_2_, medium ΔS_a_O_2_ with high base-S_a_O_2_, large ΔS_a_O_2_ with high base-S_a_O_2_, and small ΔS_a_O_2_ with low base-S_a_O_2_. There is a significant difference between the small ΔS_a_O_2_ with high base-S_a_O_2_ paradigm and all the others, except for the medium ΔS_a_O_2_ with high base-S_a_O_2_ paradigm. The pattern of *rCBV* estimations when normalizing tissue to vein is the same as in artery, but the effects of ΔS_a_O_2_, base-S_a_O_2_, and choice of contrast agent are no longer significant, as observed in the experimental data and corroborated by the simulations. The same pattern of the aforementioned parameters when normalizing to artery was also observed in the calculation of *rCBF* (Figure 7 and Supplementary Table 1). Furthermore, as shown in the experimental validations, inter-subject variability (defined as the interquartile range in the box and whisker plot) appears to be reduced as the ΔS_a_O_2_ increases and base-S_a_O_2_ decreases (Figure 6). Ultimately, the experimental and simulation data agree and show that *rCBV* and *rCBF* quantifications depend heavily on ΔS_a_O_2_ and base-S_a_O_2_, and that normalizing tissue to vein as opposed to artery results in more accuracy and consistency in the quantification of *rCBV* across different dOHb paradigms.

Of note, the pattern of *rCBV* and *rCBF* estimation for different paradigms when normalizing tissue to artery was consistent in all subjects. When normalizing to vein, the pattern was consistent in 5 out of 6 subjects; in the sixth subject, the ΔS_a_O_2_ and base-S_a_O_2_ pattern appeared to reverse when normalizing to vein, although the quantifications were not significantly different.

**Figure 8:**
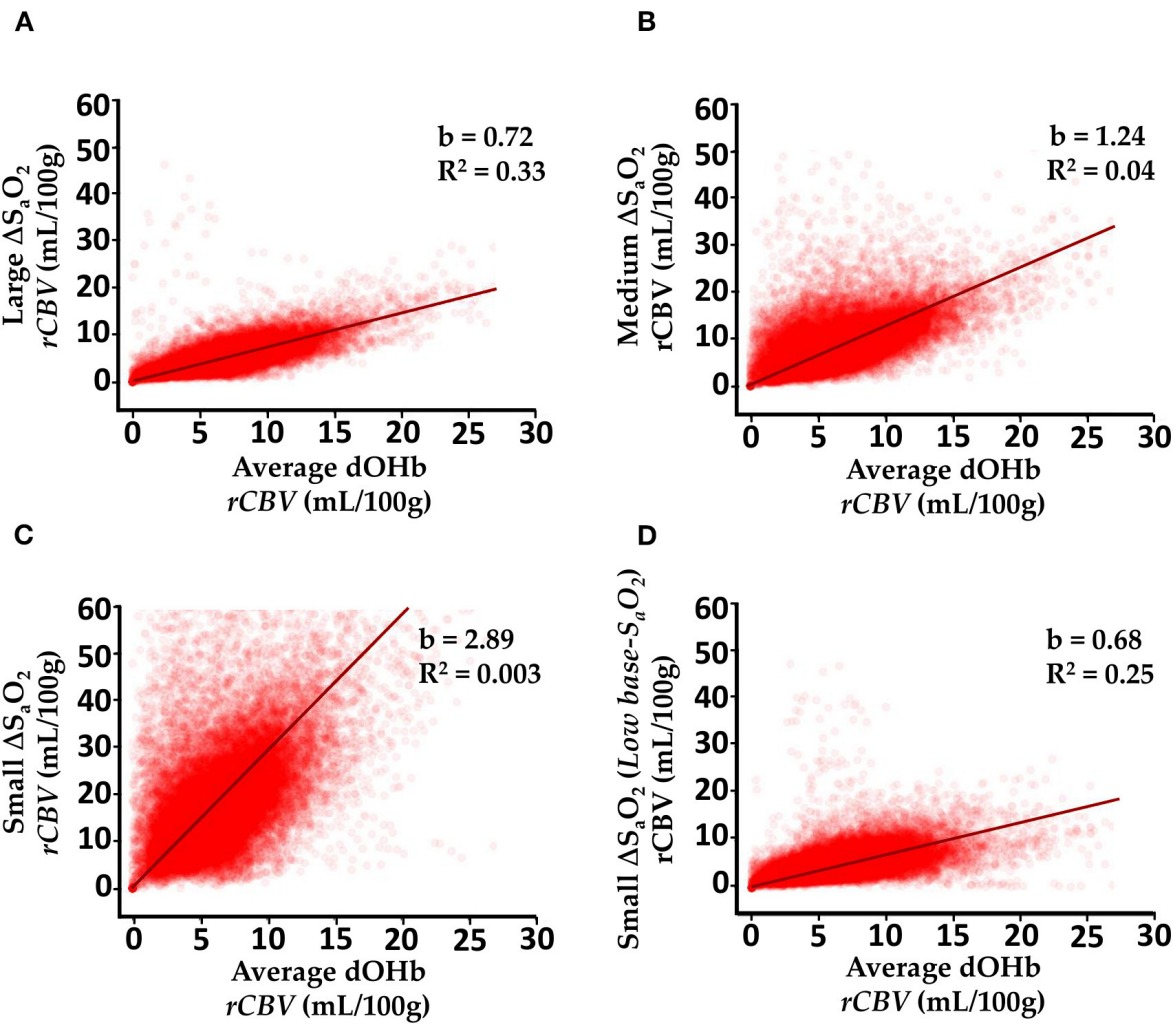
Voxel-wise Comparison of Experimentally Calculated *rCBV* (mL/100g) From dOHb Paradigms. **(A)** Large ΔS_a_O_2_ dOHb vs. average dOHb. **(B)** Medium ΔS_a_O_2_ dOHb vs. average dOHb. **(C)** Small ΔS_a_O_2_ dOHb vs. average dOHb. **(D)** Low base-S_a_O_2_ dOHb vs. average dOHb. Each plot displays data from all GM and WM voxels in a single subject. b is the regression slope and R^2^ is the coefficient of determination. Note that for this analysis specifically, *rCBV* maps were spatially smoothed with a 3x3 gaussian filter. The average dOHb paradigm is simply an average of the small, medium, and large ΔS_a_O_2_ paradigms.

Figure 8 elaborates further on the results described in Figures 5, 6 by showing voxel-wise regressions for each of the dOHb paradigms in a representative subject. The maps show all voxels within the GM and WM masks (Supplementary Figure 4). For these figures, the *CBV* maps from each dOHb paradigm were registered to the average dOHb paradigm, and then spatially smoothed with a 3 × 3 gaussian kernel. The results show that the slope of the fitted regression decreases as ΔS_a_O_2_ increases and base-S_a_O_2_ decreases, in agreement with the patterns in Figure 6. The calculated R^2^-score is very low in the paradigms with lower *SNR* but increases as the ΔS_a_O_2_ increases and base-S_a_O_2_ decreases, which is expected.

**Figure 9:**
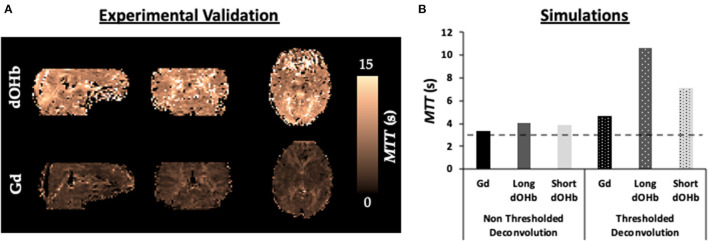
*MTT* Dependency on Bolus Duration. **(A)** Validations. *MTT* maps from a representative subject when exposed to a hypoxic bolus of roughly 30 s duration vs. a Gd bolus of roughly 10 s duration. **(B)** Simulations. Calculated *MTT* with either no thresholding (solid) or standard deconvolution thresholding (dotted) for dOHb and Gd contrast (no noise). Simulated dOHb bolus is either longer (dark gray) or shorter (light gray) to match the duration of a standard Gd bolus (black). The ground truth *MTT* is represented by the black dotted line (3 s)—anything above is an overestimation of the true *MTT*.

Figure 9 displays simulation and experimental results for *MTT* calculated with dOHb and Gd. Surprisingly, for the experimental data, the estimated *MTT* values were much greater when using dOHb than when using Gd contrast. This was observed in all dOHb paradigms for all subjects, as displayed in Supplementary Table 1. While absolute values of calculated *MTT* differed in GM and WM between Gd and dOHb, the ratio of GM to WM was more consistent, regardless of the applied contrast agent or paradigm. The simulations show the same pattern as the experimental data, with dOHb contrast resulting in higher calculated *MTT* than Gd. We noted that this is especially the case when the singular values are thresholded in deconvolution, a necessary step in removing noise prior to *rCBF* and *MTT* estimation as described in the Methods. When thresholded, a dOHb bolus of longer duration results in a higher *MTT* estimation than a dOHb or Gd bolus of shorter duration. In sum, thresholding the singular values for deconvolution, a necessary step in the experimental data analysis, and applying a bolus of longer duration both result in a greater overestimation of the *MTT*.

## Discussion

DSC MRI is widely used to determine perfusion maps in cerebral tissue. In the current study, we extend previous investigations on the accuracy and precision of perfusion quantification using Gd (references in Calamante, 2013) to the lower susceptibility range, using dOHb as contrast agent. Specifically, we investigate the influence of the following parameters on perfusion quantification: the susceptibility of contrast agent, the amount of contrast agent, baseline oxygen saturation, duration of the bolus, and choice of reference voxel (i.e., artery or vein). To address these questions, we developed a signal framework for DSC MRI that incorporates signal contributions from intravascular and extravascular water proton spins at 3T for arterial, venous, and cerebral tissue voxels. We compared the simulated predictions to experimental data obtained at 3T on six healthy human subjects administered Gd and dOHb boluses in separate acquisitions. Our major findings in the simulations and experimental validations are as follows:

- Reducing the simulated arterial *CBV* from a higher value (80-100%) to a moderate value (40-60%) reduces the estimated values of tissue *rCBV* and *rCBF*.
- Reducing the baseline S_a_O_2_, increasing the susceptibility of the applied contrast agent (Gd vs. dOHb), and/or increasing the ΔS_a_O_2_ increases inter-subject precision and reduces the estimated values of tissue *rCBV* and *rCBF*.
- Normalizing tissue to a venous rather than arterial relaxation rate time course increases the accuracy of the calculated *rCBV* and decreases quantification dependency on ΔS_a_O_2_, base-S_a_O_2_, and normalizing voxel *CBV*.
- Shortening the bolus duration increases the accuracy and reduces the estimated values of tissue *MTT*.

Please note that the aim of this work was not to optimize imaging protocols in DSC MRI; it was similarly not our goal to determine the preferred contrast agent for imaging. Rather, we used our model and experimental validations to study how different susceptibility parameters affect the precision and accuracy of perfusion quantification.

### Signal modeling framework

The signal model applied in this work was previously used to simulate fMRI signal changes in response to neuronally-driven blood oxygenation changes (Uludag et al., 2009). Given that the dOHb bolus used in this study is an externally driven susceptibility change of the same contrast agent as in fMRI, we applied the same biophysical model to DSC MRI. We generalized the framework to work for Gd contrast as well. To do so, the relationship between Gd concentration and susceptibility changes had to be determined. In doing so, we found that at 3T, a 1 mM increase of Gd is approximately equivalent in susceptibility to a 24.6% increase in dOHb (Eq. 8.2). Thus, Gd was modeled in the same way as dOHb, with differences only in the input bolus shape, duration, and susceptibility value (Supplementary Figures S1, S6). Consequently, any difference in calculated physiological parameters (e.g., *CBV, CBF* and *MTT*) can be attributed to differences in susceptibility and bolus properties.

Our model calculates the DSC MRI signal response to contrast agent in tissue, which results from both intravascular and extravascular relaxation rate changes in separate vascular compartments: artery, vein, capillary, arteriole, and venule. This has theoretical advantages over previous *in vivo* and simulation work which models DSC MRI through a “bulk model”, wherein there is no theoretical separation between extravascular and intravascular compartments (e.g., see Calamante et al., 2000; Patil and Johnson, 2011; Chappell et al., 2015). The contrast agent's influence on the proton spins in these compartments is not identical and should be treated as separate signal contributions (Zhao et al., 2007; Blockley et al., 2008; Uludag et al., 2009). The model described in Kiselev et al. incorporates extravascular and intravascular signal contributions and notes differences in the calculated and ground truth *CBV* and *CBF* of simulated voxels (Kiselev, 2001). A major difference between our work and Kiselev's is that the latter assumes an intravascular relaxation rate that is linearly dependent and an extravascular relaxation rate that is quadratically dependent on contrast concentration—our model assumes a linear extravascular and quadratic intravascular relationship.

Numerous experimental and theoretical papers report quadratic dependencies between contrast agent (dOHb or Gd) and the intravascular relaxation rate (Jensen and Chandra, 2000; Silvennoinen et al., 2003; van Osch et al., 2003; Zhao et al., 2007; Blockley et al., 2008). As a linear intravascular relaxation rate in our model would result in little to no dependency of the perfusion metrics on the susceptibility value, our experimental results are also a strong argument for a non-linear relationship between the contrast agent dose and intravascular relaxation rate. This is because the effects of contrast dosage, for example, would mostly cancel out in a linear relationship, resulting in little dependency. Regarding the extravascular relaxation rate, there is strong agreement based on rigorous Monte Carlo simulations that it is linearly related to the concentration of contrast agent (Boxerman et al., 1995; Kjølby et al., 2006; Uludag et al., 2009).

To the best of our knowledge, two studies conducted by Kjølby et al. (2006, 2009) are the only ones to account for linear extravascular and quadratic intravascular relaxation rates in DSC MRI simulations. The paper in 2006 (Kjølby et al., 2006) focused on deriving tissue relaxivity in response to Gd and did not quantify perfusion. The paper in 2009 (Kjølby et al., 2009) set out to quantify perfusion in response to changes in AIF partial volume. However, this work neglected separate vascular compartments in tissue. Therefore, even though components of our model have been proposed in some studies, the integration of all these components is novel, that is, incorporating quadratic intravascular and linear extravascular components from separate vascular compartments. In addition, the current work has investigated perfusion quantification more systematically and for a wider range of properties than previous studies.

Furthermore, it was not our aim to investigate perfusion quantification dependent on specific tissue properties, such as vessel branching, cellular packing, or vessel permeability; we instead focused on properties related to the applied contrast agents and intravascular volume fraction. Thus, we refer the reader to other DSC MRI signal models which have been developed to understand the effects of various tissue parameters (i.e., vessel heterogeneity, contrast agent extraversion) on signal and relaxation rate (Quarles et al., 2009; Semmineh et al., 2014; Digernes et al., 2017).

### Signal scaling differs between Gd and dOHb

While Gd-induced signal changes are larger than those induced by dOHb, signal scaling is not homogeneous in all areas of the brain (Figure 4). When the Δ*S*_*dOHb*_ map is divided by the Δ*S*_*Gd*_ map, termed the Δ*S* ratio, we found a significant scaling difference between arterial, venous, GM, and WM regions. Specifically, large vessel voxels, on average, show a larger Δ*S* ratio than tissue voxels in both the simulations and experiments. The likely reason for this is due to non-linear signal scaling. When signal in the large vessels approaches zero, also known as saturation (Ellinger et al., 2000), signal change becomes non-linear with respect to dosage (doubling the dosage or *CBV* results in much smaller signal changes). Given that signal in response to Gd contrast can approach the noise floor, something that does not occur with a typical hypoxic bolus (Poublanc et al., 2021; Vu et al., 2021), there is an inherent difference in how signal is scaled between contrast agents with high vs. low susceptibility in large vessels. The scaling is more linear for dOHb and more non-linear for Gd, with respect to the amount of contrast agent.

Although to a much smaller degree, there is also a scaling difference between the GM and WM voxels, wherein the GM Δ*S* ratio between Gd and dOHb induced changes is lower than the WM Δ*S* ratio. This effect is observed in the experiments but not in the simulations (Figure 4). One potential reason for the observed difference between GM and WM might be macrovascular contamination (Chappell et al., 2015), particularly the so-called blooming effect (reviewed by Willats and Calamante, 2013). It is known that the administration of Gd results in signal contamination in the cortical GM, where magnetic field disturbances from large vessels extends to the surrounding tissue (reviewed by Willats and Calamante, 2013). Assuming that these vessels are large arteries, this effect would be largely scaled down when using dOHb contrast due to the far lower signal arising from the arterial vessels on the cortical surface (refer to Figures 2, 3). Further work would be required to isolate for and identify the magnitude of the blooming effect when using Gd and dOHb. In summary, the signal change induced by Gd is larger than that induced by dOHb, but the scaling is not uniform throughout the brain, confirmed both in the experiments and simulations. This is a novel finding, as no previous work has compared signal change maps obtained from contrast agents with vastly different molar susceptibilities.

Finally, the Δ*S* ratio in arterial voxels is lower than that in venous voxels for both the simulations and experimental data. The reason for this is more complicated than the prior findings and can best be understood by referring to the simulation findings in Figure 3A. Here, although results are displayed for Δ*R*${}_{2,max}^{*}$ as opposed to Δ*S*_*max*_, the same rationale applies. The difference between Gd and dOHb is magnified in arterial voxels and is much less apparent in venous voxels—the division of Δ*S*_*max, dOHb*_ by Δ*S*_*max, Gd*_ therefore yields much lower values in arterial voxels. The mathematical reasoning for the difference between artery and vein will be discussed further in the following sections.

### Calculated *rCBV* and *rCBF* depend on simulated *CBV* of normalizing voxel, ΔS_*a*_O_2_, and base-S_*a*_O_2_

The simulations and experiments show a dependence of *rCBV, rCBF*, and *MTT* on user-controlled parameters, such as the type of contrast agent, choice of normalizing voxel, ΔS_a_O_2_, base-S_a_O_2_, and bolus duration. Figure 3 and Supplementary Figure 5 show that the area and magnitude of the arterial bolus relaxation curve at 3T do not increase linearly as the simulated blood volume increases, but rather increase and then decrease at higher simulated *CBV* occupancy. This counterintuitive pattern is also observed in previous work with Gd, where simulated arterial voxels with 80% *CBV* showed a greater signal change than those with 100% *CBV* (Kjølby et al., 2009). It is the predominant intravascular component of simulated arterial and venous voxels that results in a non-linearity in *CBV* dependence (Supplementary Figure 7); for simulated tissue voxels, this non-linearity is not observed due to the predominant linear extravascular relaxation rate. As such, the model predicts that normalizing tissue voxels to an arterial voxel with high blood volume results in higher calculated *rCBV* values than when normalized to an arterial voxel with an intermediate blood volume (Supplementary Figure 7).

As shown in Figures 5–8 and Supplementary Table 1, *rCBV* and *rCBF* attained with dOHb becomes more accurate and decreases as the bolus's ΔS_a_O_2_ increases and base-S_a_O_2_ decreases. Experimentally, this pattern should be more predominant when tissue is normalized to an arterial voxel with a high *CBV*, as from the theory and simulations, it is primarily the intravascular quadratic relaxation rate that is responsible for these dependencies (Supplementary Figure 7). The concept of the intravascular space contributing to perfusion overestimation is also acknowledged in previous works (Kjølby et al., 2009; Wirestam et al., 2010). As mentioned above, we show that increasing the susceptibility of contrast agent decreases the calculated *rCBV* and *rCBF*, without the confounds of saturation or partial volume errors in the case of our simulations. This pattern is more evident when using a lower susceptibility contrast agent, such as dOHb, which likely explains why previous researchers investigating the dosage of Gd found only a small effect of dosage on quantification (Alger et al., 2009; Wirestam et al., 2010).

Ultimately, the large deviations in the estimation of *rCBV* and *rCBF* between different dOHb and Gd paradigms are expected in accordance with the simulations. Of note, the *rCBF* values obtained in this work overestimate those from previous work using dOHb, which in turn overestimate physiological values determined by PET (Knutsson et al., 2010; Vu et al., 2021). This is similarly not unexpected as previous work applied a different experimental paradigm, which we now know from our simulations and validations will lead to a differing calculation of *rCBF*.

As an additional note, Bleeker et al. showed that it is beneficial to select an AIF adjacent to the intravascular space of an artery (Bleeker et al., 2009). Our work supports this claim for two reasons: firstly, our simulations show that normalizing tissue to an AIF with a low to intermediate simulated *CBV* results in higher accuracy of the calculated *rCBV;* secondly, our simulations reveal a lower dependence of the calculated *rCBV* on ΔS_a_O_2_ and base-S_a_O_2_ when tissue is normalized to a less intravascular AIF (Supplementary Figure 7).

### Calculated *rCBV* depends on the choice of artery or vein for tissue normalization

From a clinical perspective, it might be beneficial to normalize tissue signal to a venous as opposed to arterial voxel, as the larger radius of the venous blood vessel results in less partial volume errors (reviewed by Willats and Calamante, 2013). Although there are still limitations to this type of analysis in DSC MRI (Knutsson et al., 2007), we sought to investigate how perfusion quantification depends on arterial vs. venous normalization.

In simulations and experiments, normalizing $\Delta R_{2,tissue}^{*}\left(t\right)$ to $\Delta R_{2,vein}^{*}\left(t\right)$ results in lower overestimations of *rCBV*, along with a reduced ΔS_a_O_2_ and base-S_a_O_2_ dependency in comparison to when $\Delta R_{2,tissue}^{*}\left(t\right)$ is normalized to $\Delta R_{2,artery}^{*}\left(t\right)$ (Figures 5, 6, and Supplementary Figure 5). As previously described, a lower base-S_a_O_2_ results in larger relaxation rate changes on account of the quadratic intravascular dependence on [Gd] and SO_2_, which then influences perfusion estimation. Thus, if a vein and an artery are provided the same ΔS_a_O_2_ or [Gd] and are simulated with the same *CBV*, it is clear from the previous results that the voxel with a lower base-S_a_O_2_, as is the case in veins, will have a larger relaxation rate change (specifically when the voxel is mostly intravascular and when the contrast agent has a lower susceptibility or concentration). This theoretical discovery likely explains the high venous to arterial relaxation rate ratio observed in previous studies using dOHb in DSC MRI (Poublanc et al., 2021; Vu et al., 2021). When tissue signal is then normalized to the venous voxel with a larger relaxation rate change relative to artery, and thus, larger area under curve, a lower and more accurate estimate of *rCBV* is obtained. When normalizing to a vein, we found that varying the ΔS_a_O_2_ and base-S_a_O_2_ through different paradigms had less of an effect on perfusion quantification in both the simulations and experimental validations. A reduced contrast dosage dependency and lower calculated *rCBV* when normalizing to vein as opposed to artery is also observed in previous experimental results using Gd (Wirestam et al., 2010).

### Calculated *MTT* and *rCBF* depend on bolus duration

The experimental data reveal an overestimation of the calculated *MTT* in every participant when using dOHb in comparison to Gd for quantification (Figure 9). Vu et al. also reported high calculated *MTT* values when using dOHb as a contrast agent in a standard DSC MRI analysis (Vu et al., 2021).

The simulations reveal that bolus duration influences *MTT* and *rCBF* calculation (Figure 9). We also found that the thresholding of singular values causes a duration dependency—longer boluses resulted in the removal of more singular values from the AIF. It is known that as more singular values are removed, there is a greater underestimation of *rCBF* and, therefore, greater overestimation of *MTT* (Bjørnerud and Emblem, 2010). Thus, a Gd bolus with shorter duration and less removed singular values results in the calculation of a lower and more accurate *MTT* relative to the *MTT* calculated from a dOHb bolus. In our simulation work, when the dOHb bolus is shortened to the duration of Gd, the calculated *MTT* is more like that obtained with Gd (Figure 9). In previous work where Gd was doubled in concentration, an increase in the calculated *MTT* was found relative to a standard dose (Wirestam et al., 2010). Another study found that reducing the injection rate of Gd results in an increase to the calculated *MTT* (van Osch et al., 2003). As the bolus had increased in duration for these two studies, our findings support and help explain the observations.

Although noise was not simulated in our work, it is known that *SNR* is another major parameter influencing *MTT* calculation, wherein more singular values from the AIF matrix are removed when there is a lower *SNR*, resulting in a greater underestimation of *rCBF* and overestimation of *MTT* (Willats and Calamante, 2013). This may explain why the different dOHb paradigms in our work, although equal in duration, show an increasing estimation of *MTT* as the *SNR* decreases (Supplementary Table 1). Thus, a combination of low *SNR* and long bolus duration leads to higher estimations of *MTT*, supporting the finding that a dOHb bolus yields a higher *MTT* estimation than a Gd bolus.

### Considerations for clinical implementation of dOHb and Gd

For dOHb contrast to attain an *SNR* like that of Gd in a single bolus, the oxygen saturation must drop below physiological tolerance (Supplementary Figure 1 and Supplementary Table 1). However, DSC using dOHb has several advantages (Poublanc et al., 2021; Vu et al., 2021). Firstly, as dOHb is an endogenous contrast agent, it does not share the safety limitations associated with Gd (Schlaudecker and Bernheisel, 2009; Voth et al., 2011; Kanda et al., 2015; Lord et al., 2018; Rogowska et al., 2018). As well, [dOHb] does not saturate, recirculate, or accumulate, as the blood oxygenation is reset to a pre-determined value in the lungs using the RespirAct^®^. Unlike Gd, the concentration of dOHb in the arterial blood can be determined from end-tidal gas analysis which may be useful as a precise AIF and aid in quantitative modeling (Ito et al., 2008; Fierstra et al., 2011). Finally, dOHb acquisitions can be repeated and averaged to improve the *SNR* (“Average dOHb” in Supplementary Table 1).

While the smaller hypoxic challenge (ΔS_a_O_2_ of 8%) administered in our study is equivalent to experiencing reduced S_a_O_2_ in moderately high altitudes (e.g., Aspen, Colorado), the larger challenge (ΔS_a_O_2_ of 24%) is closer to the S_a_O_2_ in the Everest Base Camp (Rojas-Camayo et al., 2018), albeit only for a short duration. Individuals may spend many months in these environments without additional oxygen or altitude sickness, minimizing the safety concern in our study where hypoxia only lasts for roughly 30 s.

### Limitations and directions

We have not included T_1_ effects in our simulations. According to Calamante et al., a TR of 1.5 s and flip angle of 73° would result in, roughly, a 10% underestimation of *CBF* relative to that calculated in the absence of T_1_ effects, although this depends on the inflow rate (Calamante et al., 2007). This is to be considered in future studies but is not of major concern in our work as we are not investigating absolute values, but rather how relative quantification depends on various paradigms. It is not expected that the general patterns of the findings will change by including T_1_ effects, especially in the absence of contrast agent extraversion. As well, it has been shown in previous work that there is no significant T_1_ change in response to changes in dOHb, limiting the T_1_ effect concern to Gd (Thulborn et al., 1982). A further limitation of our study and DSC MRI theory in general is the correct simulation of [Gd]_max_. Studies have reported varying quantities for [Gd]_max_ (Patil et al., 2013; Kellner et al., 2018; Lind et al., 2020). Simulating the exact value of [Gd]_max_ is not a major concern in this work, but it should be noted that *in vivo*, a standard Gd dose likely results in a [Gd]_max_ that is larger than the simulated 2 mM used in the majority of our simulations.

In the model, we do not include contrast agent extravasation, which is known from previous modeling and experiments to elicit additional ${\text{T}}_{2}^{*}$ and T_1_ relaxation effects, limiting perfusion quantification accuracy (Quarles et al., 2005, 2009). Additional relaxation effects that result from leakage, dependent on the molecular size of contrast agent, can be implemented in future work that simulates pathological cerebral tissue. The limitation of neglecting this effect in our modeling is likely minimal as healthy subject tissue is assumed to have an intact blood-brain-barrier, with little to no leakage (Quarles et al., 2009).

Vasodilation from the hypoxic stimulus is another potential experimental limitation. Previous work showed that dropping S_a_O_2_ from 100 to 90% for a 20-min duration is the threshold for statistically significant cerebral vasodilation in humans (Gupta et al., 1997). According to more recent work, an S_a_O_2_ of ~70% results in increases to the *CBF* (Mardimae et al., 2012); however, the vascular response is delayed by about 3 minutes following hypoxia (Harris et al., 2013). We expect that there is no significant vasodilation in this work as the lowest S_a_O_2_ of 75% only lasts for approximately 30 s. Nevertheless, vasodilation would have to be accounted for in more drastic hypoxic paradigms, or in a diseased state, to avoid errors in quantification—this can easily be done with our model. Furthermore, for isocapnic hypoxia, as utilized in this study, no changes in the tissue oxygen metabolism or oxygen extraction fraction are expected (Ainslie et al., 2014; Gu and Jun, 2018).

As a future direction, dOHb contrast can be studied with a higher field strength scanner to further bolster *SNR*, which might be the best way to maximize the benefits of dOHb as long as the arterial signal does not become saturated. It is also known that at higher field strengths, the intravascular contribution in ${\text{T}}_{2}^{*}$-weighted acquisitions is heavily reduced relative to extravascular signal contribution (Uludag et al., 2009), which would lead to lower errors in perfusion quantification. It might be worthwhile, for a more detailed validation of the Gd to dOHb relationship determined in our modeling, to compare dOHb with low-dose Gd as contrast, where the low-dose of Gd better approximates the susceptibility induced by dOHb. We are currently in the planning stages of such a study. Another potential direction would be to investigate the use of dOHb contrast in clinical conditions, such as brain tumors, steno-occlusive vascular disease, and dementia. This could first be simulated with our framework and then validated with clinical scans. Finally, it would be useful to explore either a model-based DSC MRI analysis method or calibration approach using our DSC model, which should provide more consistent and accurate quantifications of perfusion.

## Conclusion

We developed a DSC MRI signal framework to simulate cerebral tissue signal changes in response to dOHb and Gd contrast at 3T. We used this framework to investigate how various contrast agent parameters and tissue properties influence perfusion estimation. In doing so, perfusion quantification dependencies were discovered that are in agreement between the simulations and validations. We hope our model and findings will help inform future research and clinical protocols that employ dOHb and Gd as contrast agents.

## Data availability statement

The raw data supporting the conclusions of this article will be made available by the authors, without undue reservation.

## Ethics statement

The studies involving human participants were reviewed and approved by University Health Network Research Ethics Board. The patients/participants provided their written informed consent to participate in this study.

## Author contributions

JS: involved in all areas of project, including project and methods design, simulation development, data collection, data analysis/interpretation, and writing. ES and JP involved in methods design, data collection, and writing. AM: involved in methods design, simulation development, and writing. OS: involved in data collection and writing. JD: involved in writing. JF and DM: involved in data interpretation and writing. KU: involved in all areas of project, including project and methods design, simulation development, data collection, data analysis/interpretation, and writing. All authors contributed to the article and approved the submitted version.

## Footnotes

^1^As the susceptibility values change with time (i.e., due to changes in blood oxygenation or in contrast agent concentration), the relaxation rates, MRI signal contributions, and the total signal are also a function of time. However, for simplification, we omit “time” in this and the following equations in the forward model section.

^2^In Uludag et al. (2009), *S*_*EES*_ and *S*_*IVS*_ were termed *S*_*ex*_ and *S*_*in*_, respectively.

## Supplementary material

Supplementary Figure 1 Susceptibility Relationship of Gd and dOHb in Simulations. Peak ΔS_a_O_2_ (%) and Gd (mM) as a function of peak susceptibility induced by contrast agent. The region shaded in blue is the typical peak susceptibility elicited in a simulated hypoxic bolus (Peak ΔS_a_O_2_ ≈ 10-25%). The region shaded in red is the typical peak susceptibility elicited in a simulated Gd bolus (Peak [Gd] ≈ 2-5 mM).

Supplementary Figure 2 Summary of Simulation Method. **(A)** Forward Model (top). Bolus time courses for Gd [mM] and dOHb [S_a_O_2_ (%)] are simulated; tissue, arterial, and venous properties (*CBV, MTT, CBF*) are then applied; the Δ*R*_2_${}_{EES}^{*}$ and Δ*R*_2_${}_{IVS}^{*}$ responses for water protons are modeled; finally, the resultant $\Delta R_{2}^{*}\left(t\right)$ time courses are fed into a signal model. **(B)** Inverse Solution (bottom). Tissue, arterial, and venous *S(t)* time courses are generated from Eq. 1—these are then converted into $\Delta R_{2}^{*}\left(t\right)$ (Eq. 9). The arterial input and tissue response curves are then used to calculate *rCBV, rCBF*, and the *MTT*.

Supplementary Figure 3 Visual Illustration of Workflow for Signal Model. Parameters, in red, are based on the values in Table 2. This process was used to simulate the signal and relaxation time courses for arterial, tissue, and venous voxels.

Supplementary Figure 4 GM, WM, and Large Vessel Masks Created in Functional Space. **Red**. Large vessels. **Blue**. GM. **White**. WM. For WM, voxels containing values within the lowest 15–20% of the Δ*S* data range were kept; for GM, the highest 75% were kept. The maps were binarized and linearly registered to the functional space of each dOHb paradigm. GM and WM were further masked with a thresholded ε_*t*_ map (voxels with values in the lowest 10% of data range were kept in mask) to remove noisy voxels in peripheral brain tissue, skull base, vein, and artery. The GM and WM regions were finally masked with a thresholded Gd *MTT* map (lowest 70% of robust range voxels were kept in mask) to remove voxels in the CSF. Large vessel mask (only used for results in Figure 4) generated by taking the highest 50% of Δ*S*_*Gd*_ or Δ*S*_*dOHb*_ robust range.

Supplementary Figure 5 Simulated $\Delta R_{2}^{*}\left(t\right)\;$for dOHb and Gd Contrast. **(A)** $\Delta R_{2}^{*}\left(t\right)\;$for arterial voxel with varying intravascular space in response to dOHb. (**B)** Same for Gd (bottom). **(C)** $\Delta R_{2}^{*}\left(t\right)\;$for tissue voxel with varying intravascular space in response to dOHb. **(D)** Same for Gd (bottom). **(E)** $\Delta R_{2}^{*}\left(t\right)\;$for venous voxel with varying intravascular space in response to dOHb. **(F)** Same for Gd (bottom). Note that tissue curves (*MTT* = 3 s) are convolved with a more quickly decaying exponential than venous curves (*MTT* = 4 s).

Supplementary Figure 6 Perfusion Ratios as a Function of Peak Susceptibility Induced by Contrast Agent. The region shaded in blue is the typical peak susceptibility elicited in a hypoxic dOHb bolus (Peak ΔS_a_O_2_ ≈ 10-25%). The region shaded in red is the typical peak susceptibility elicited in a Gd bolus (Peak [Gd] ≈ 2-5 mM). **(A)** *rCBV* (%) as a function of peak susceptibility induced by contrast agent. Simulated artery *CBV* = 100%; simulated tissue *CBV* = 4%. **(B)** Ratio of arterial to venous relaxation rate time integral as a function of peak susceptibility induced by contrast agent. Simulated arterial and venous *CBV* = 100%.

Supplementary Figure 7 dOHb *rCBV* as a Function of Simulated Arterial or Venous CBV (%). *rCBV* accuracy (y-axis) for tissue (*CBV* = 4%) when normalized to artery (or vein) of varying simulated *CBV* (x-axis). *rCBV* accuracy calculated as *rCBV*_*calculated*_ divided by *rCBV*_*simulated*_. All values above 1 (dotted line) overestimate the *rCBV*_*simulated*_. **(A)** Three scenarios are described in this figure: only extravascular component of signal model used in simulated artery (black plus); only intravascular component of signal model used in simulated artery (dark gray square); full signal model used in simulated artery (light gray diamond). **(B)**. Tissue data normalized to AIF (black) or VOF (gray). Four scenarios are described in this figure: large ΔS_a_O_2_ with high base-S_a_O_2_ (diamond), medium ΔS_a_O_2_ with high base-S_a_O_2_ (square), small ΔS_a_O_2_ with high base-S_a_O_2_ (plus), small ΔS_a_O_2_ with low base-S_a_O_2_ (triangle).

Supplementary Figure 8 Gd and dOHb Experimental Relaxation Rate Time Courses. **(Top)** Gd time course for a single bolus. **(Bottom)** dOHb time course for three boluses. Red represents the AIF; blue represents the VOF; dark gray represents gray matter; light gray represents white matter.

Supplementary Table 1 Summary of Perfusion Data Collected with dOHb and Gd Contrast Agents. Average and standard deviations are pooled from a total of six healthy participants (age 22–60; one female). Low baseline dOHb is only pooled from five participants due to motion.

Supplementary Table 2 Table of Parameters.
