# Supplementary material for: DSC MRI in the human brain using deoxyhemoglobin and gadolinium—Simulations and validations at 3T
Source: Front Neuroimaging. 2023 Feb 20;2:1048652. doi: 10.3389/fnimg.2023.1048652 (PMC10406263; doi:10.3389/fnimg.2023.1048652)
Supplement: Supplementary Figure 1 — Susceptibility Relationship of Gd and dOHb in Simulations. Peak ΔSaO2 (%) and Gd (mM) as a function of peak susceptibility induced by contrast agent. The region shaded in blue is the typical peak susceptibility elicited in a simulated hypoxic bolus (Peak ΔSaO2 ≈ 10-25%). The region shaded in red is the typical peak susceptibility elicited in a simulated Gd bolus (Peak [Gd] ≈ 2-5 mM). [file Presentation_1.PPTX]

## Slide 1
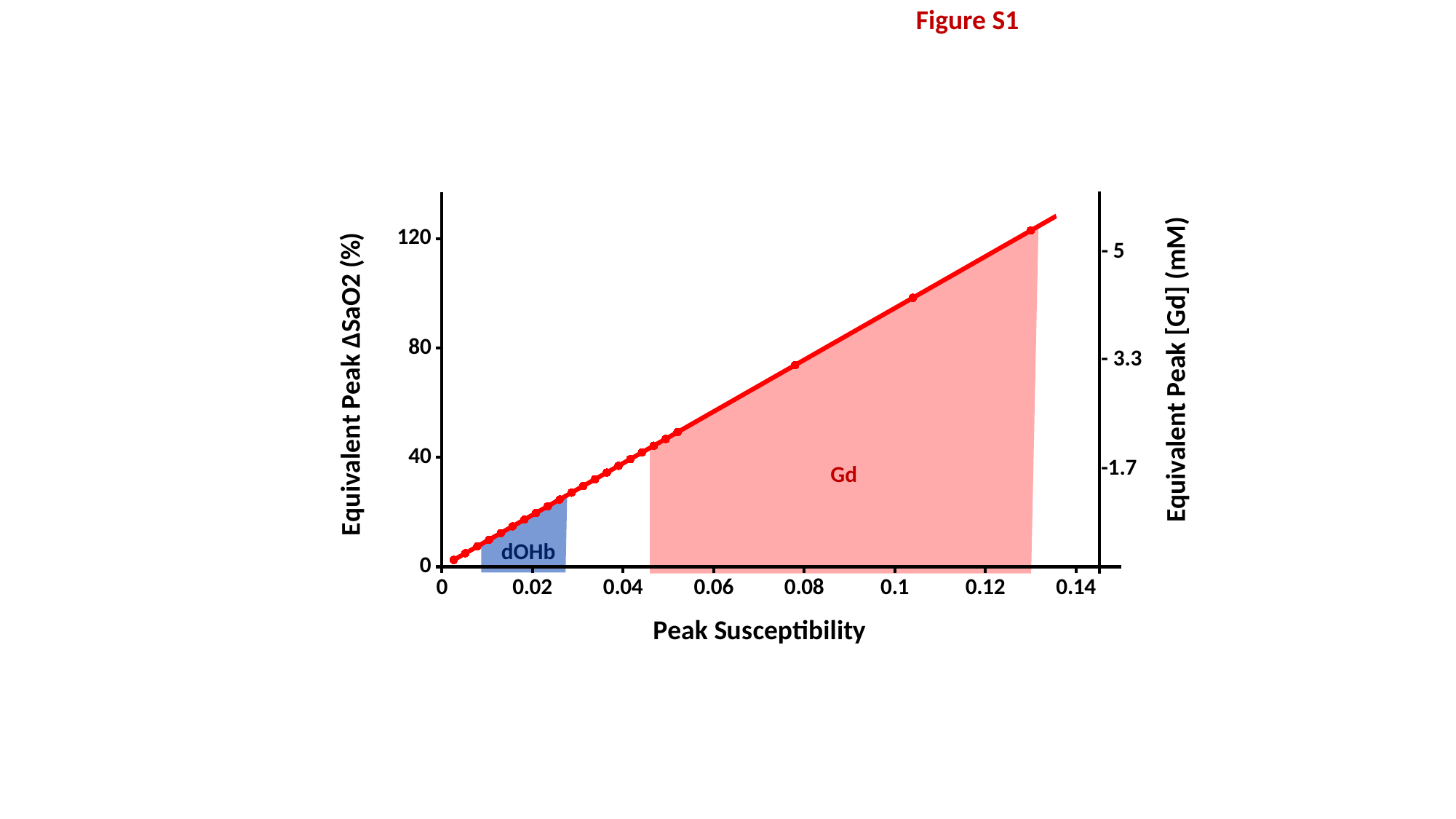

Figure S1
### Chart
| Category | Equivalent Peak ∆SaO2 (%) |
|---|---|
- 5
- 3.3
Equivalent Peak [Gd] (mM)
-1.7
Gd
dOHb

## Slide 2
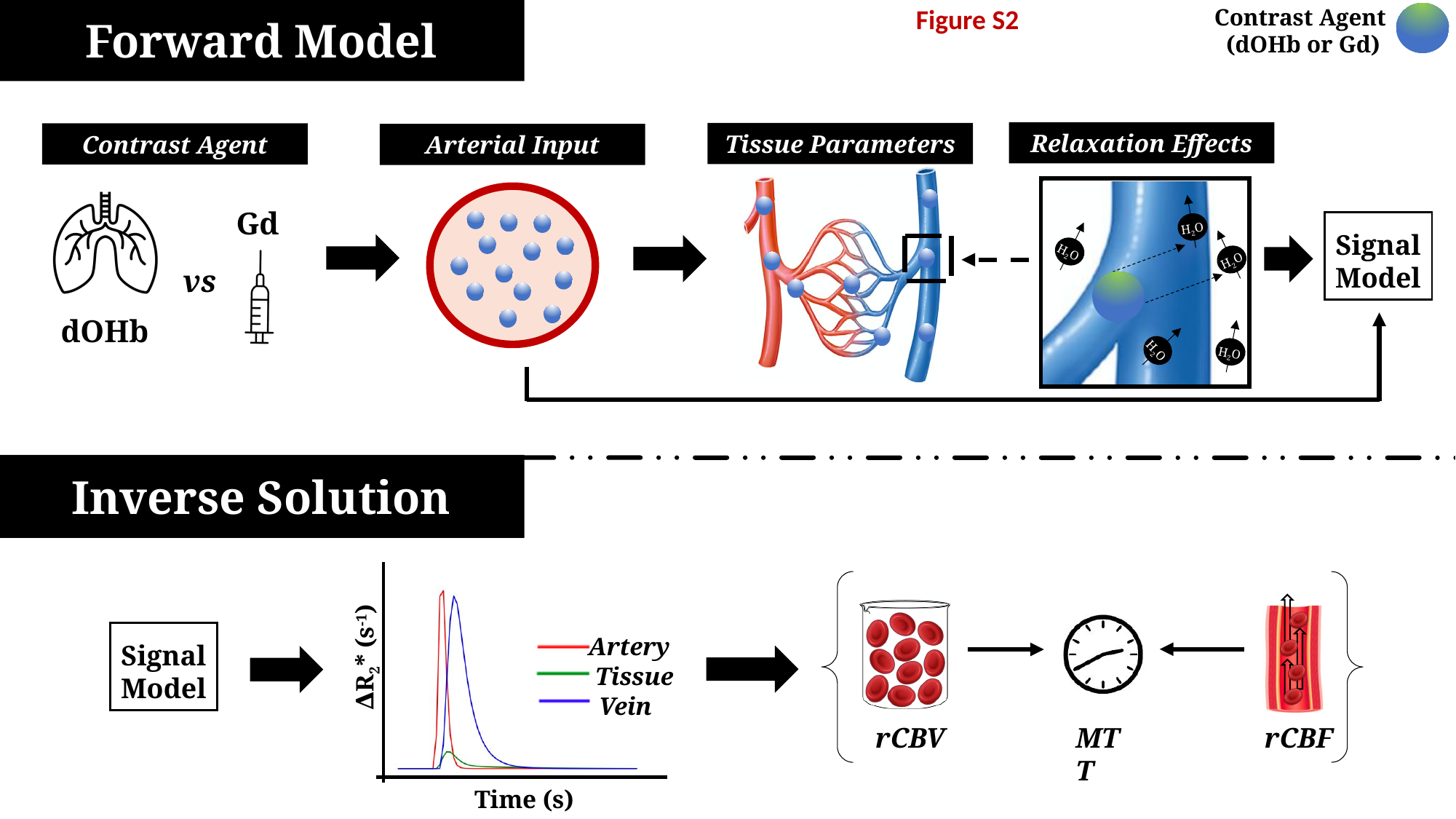

Figure S2
Contrast Agent
(dOHb or Gd)
Forward Model
Relaxation Effects
Tissue Parameters
Contrast Agent
Arterial Input
H2O
Gd
H2O
Signal Model
H2O
vs
dOHb
H2O
H2O
Inverse Solution
Artery
Signal Model
∆R2* (s-1)
Tissue
Vein
rCBV
MTT
rCBF
Time (s)

## Slide 3
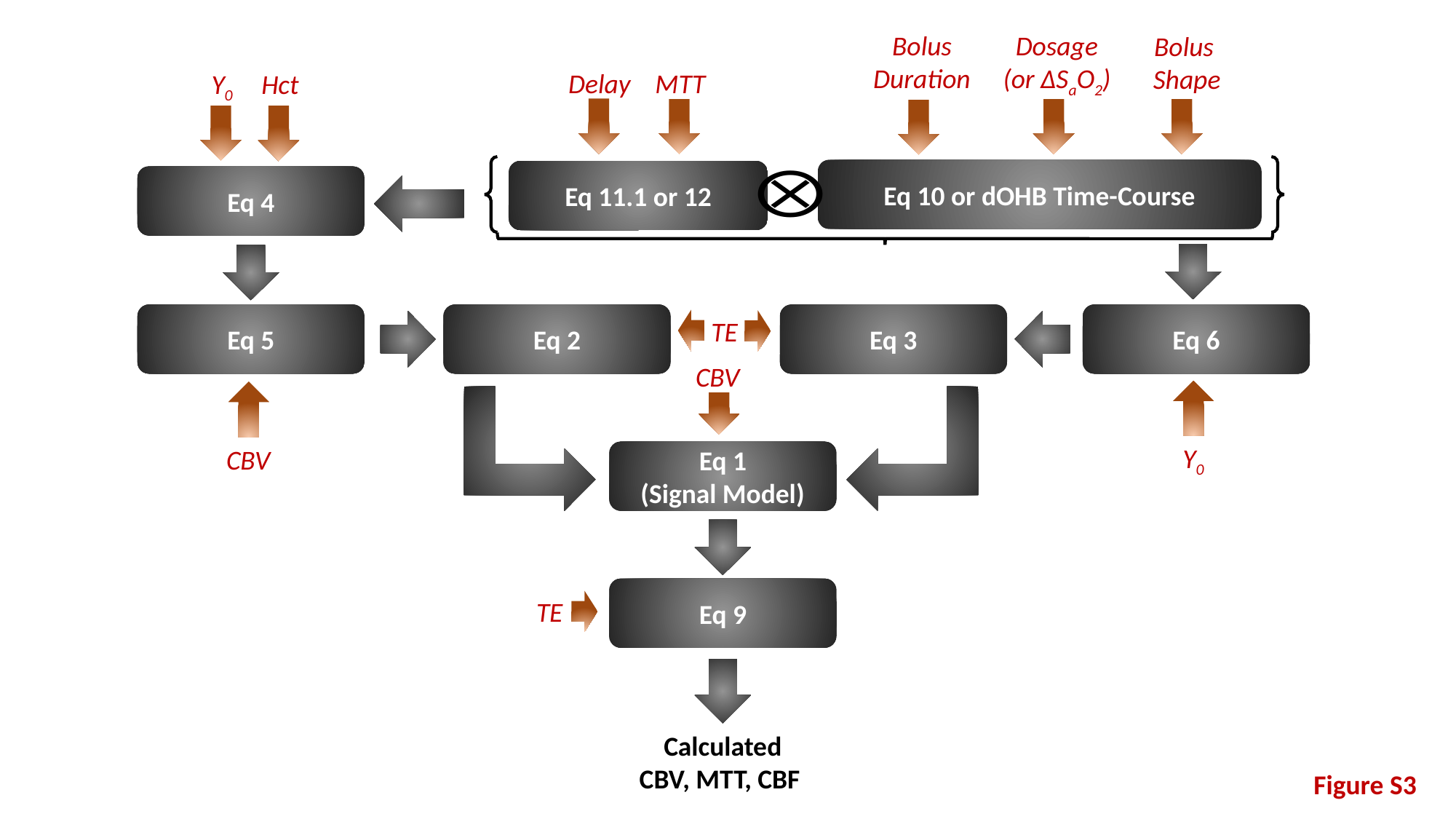

Bolus Duration
Dosage
(or ∆SaO2)
Bolus
Shape
Delay MTT
Y0
Hct
Eq 10 or dOHB Time-Course
Eq 11.1 or 12
Eq 4
Eq 5
Eq 2
Eq 6
Eq 3
TE
CBV
Y0
CBV
Eq 1
(Signal Model)
Eq 9
TE
Calculated CBV, MTT, CBF
Figure S3

## Slide 4
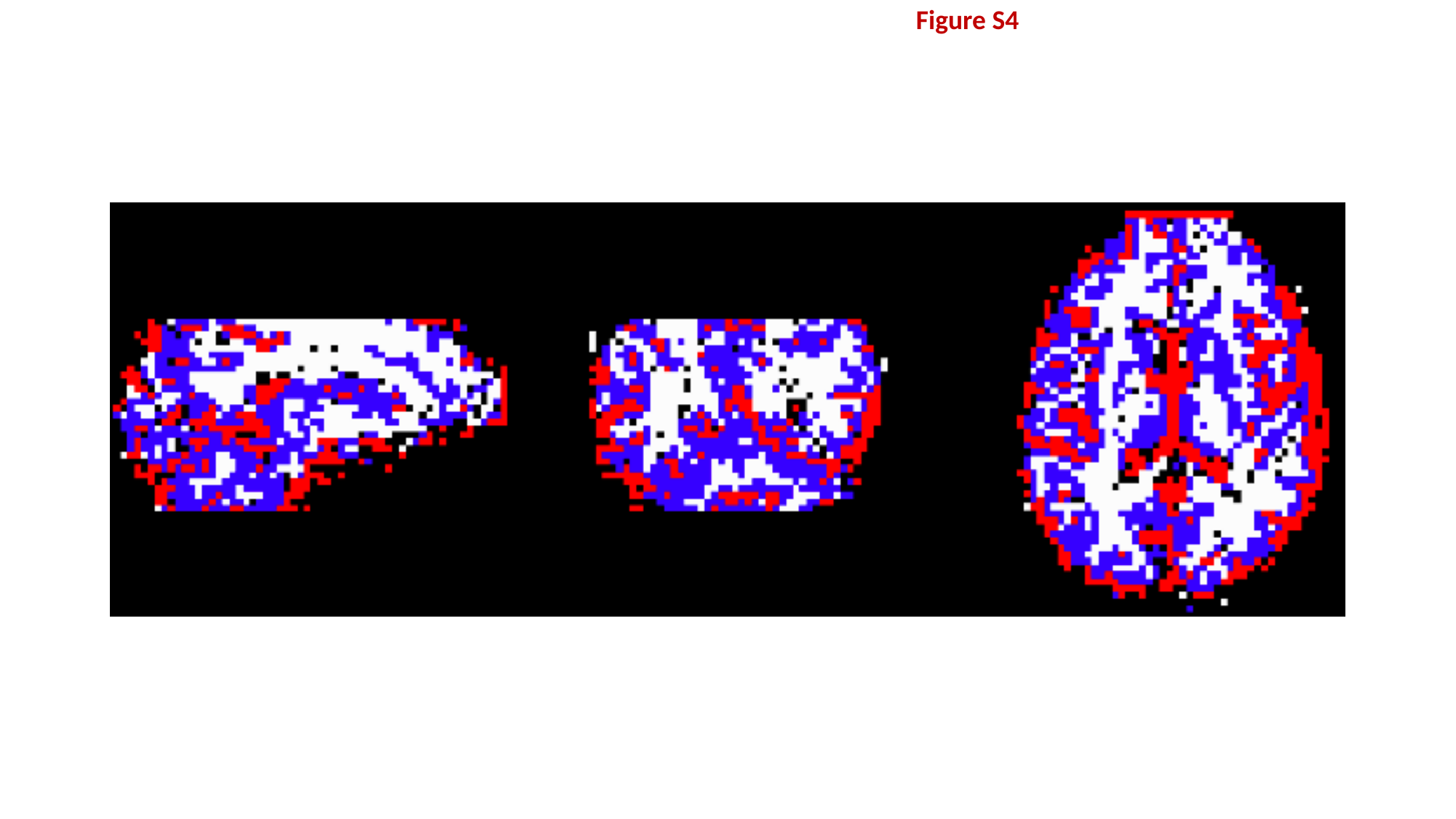

Figure S4

## Slide 5
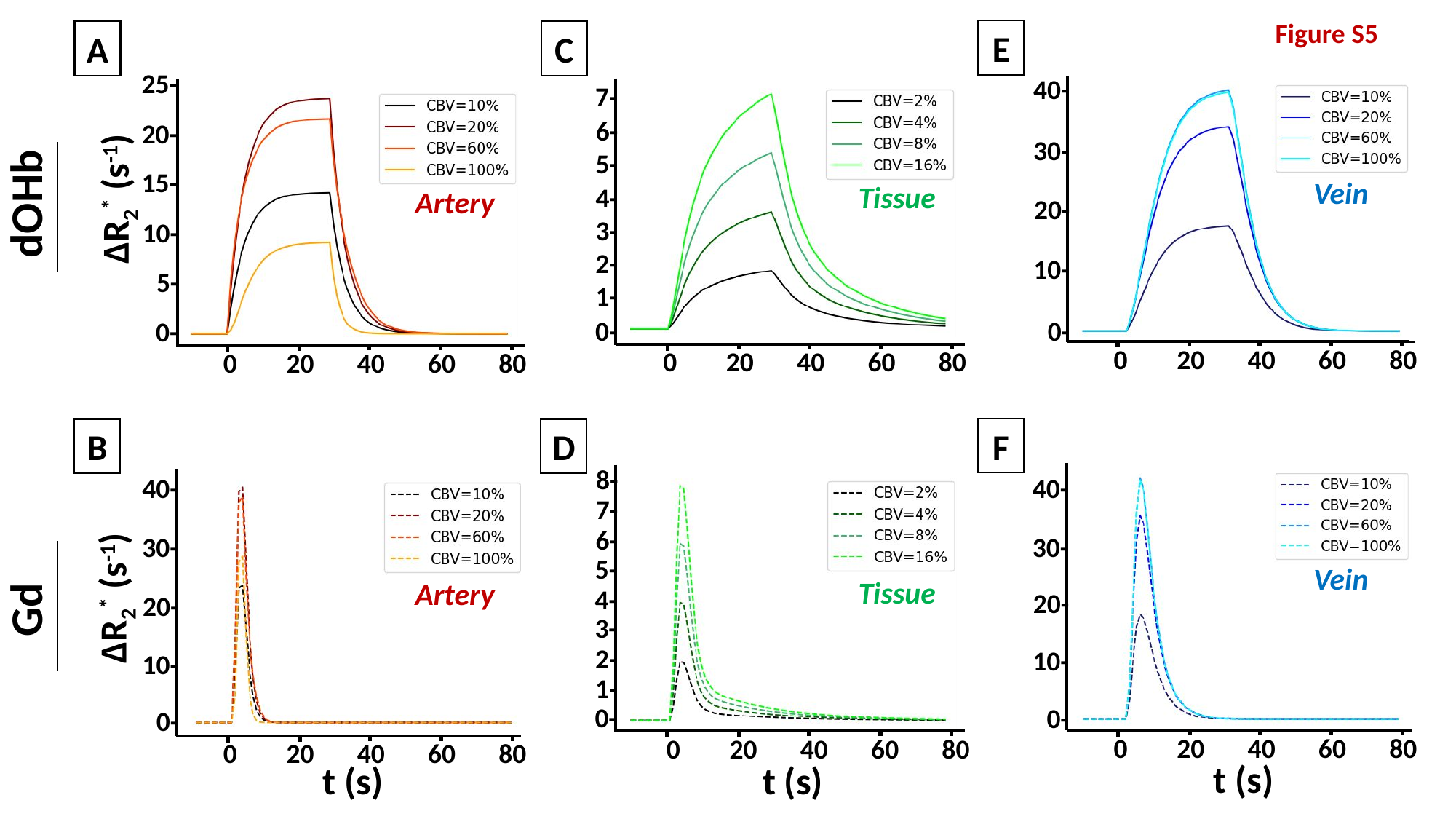

Figure S5
E
A
C
25-
40-
7-
6-
20-
30-
5-
15-
Vein
∆R2* (s-1)
dOHb
∆R2* (s-1)
Tissue
4-
Artery
20-
3-
10-
2-
10-
5-
1-
0-
0-
0-
0 20 40 60 80
0 20 40 60 80
0 20 40 60 80
F
B
D
8-
40-
40-
7-
6-
30-
30-
5-
Vein
Tissue
∆R2* (s-1)
Artery
∆R2* (s-1)
Gd
4-
20-
20-
3-
2-
10-
10-
1-
0-
0-
0-
0 20 40 60 80
0 20 40 60 80
0 20 40 60 80
t (s)
t (s)
t (s)

## Slide 6
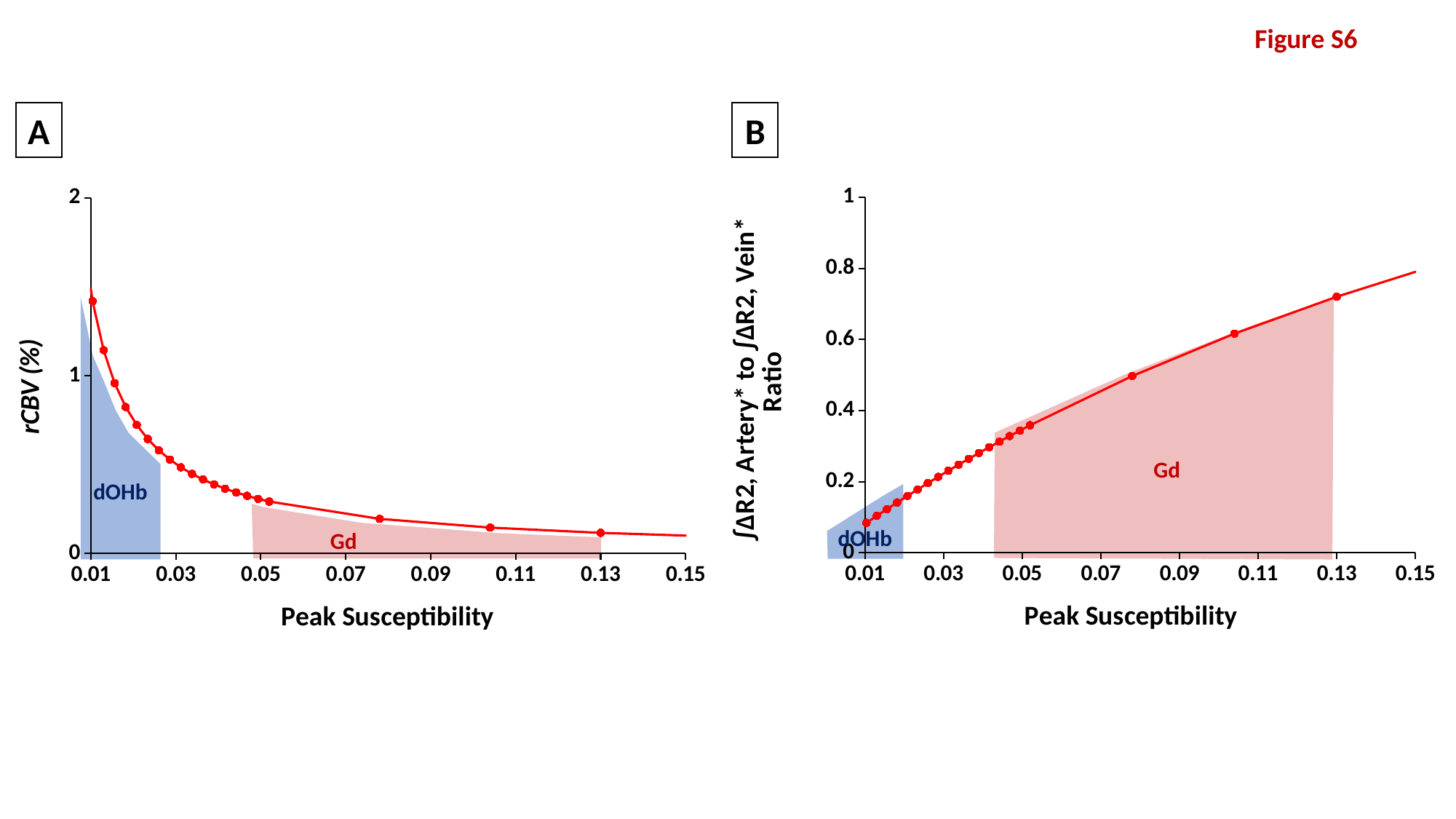

Figure S6
A
B
### Chart
| Category | ∫Arterial to ∫Venous Ratio |
|---|---|
### Chart
| Category | CBV |
|---|---|
dOHb
Gd
dOHb
Gd

## Slide 7
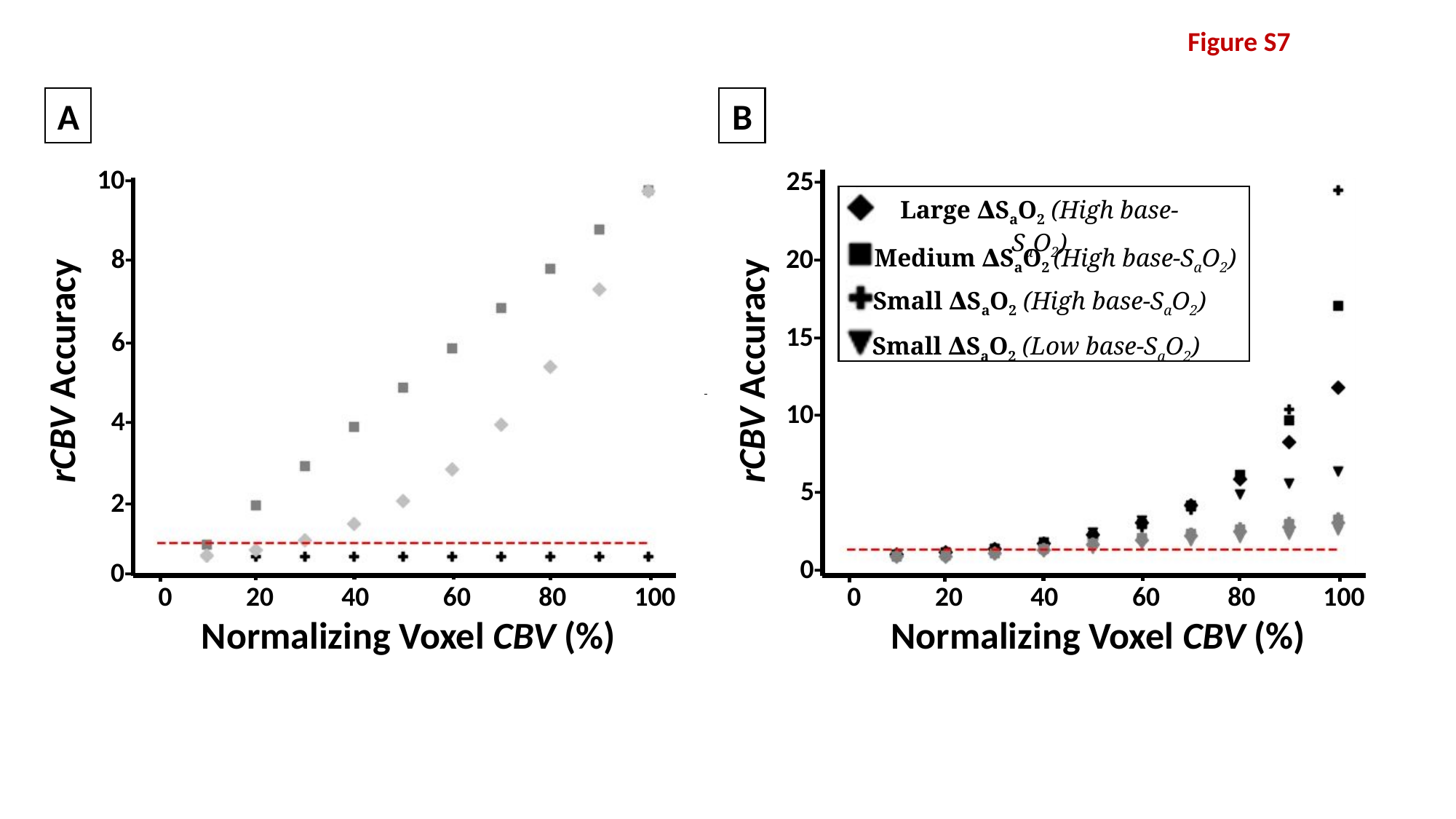

Figure S7
A
B
10-
25-
Large ∆SaO2 (High base-SaO2)
20-
8-
Medium ∆SaO2 (High base-SaO2)
Small ∆SaO2 (High base-SaO2)
15-
6-
Small ∆SaO2 (Low base-SaO2)
rCBV Accuracy
rCBV Accuracy
10-
4-
5-
2-
0-
0-
0 20 40 60 80 100
0 20 40 60 80 100
Normalizing Voxel CBV (%)
Normalizing Voxel CBV (%)

## Slide 8
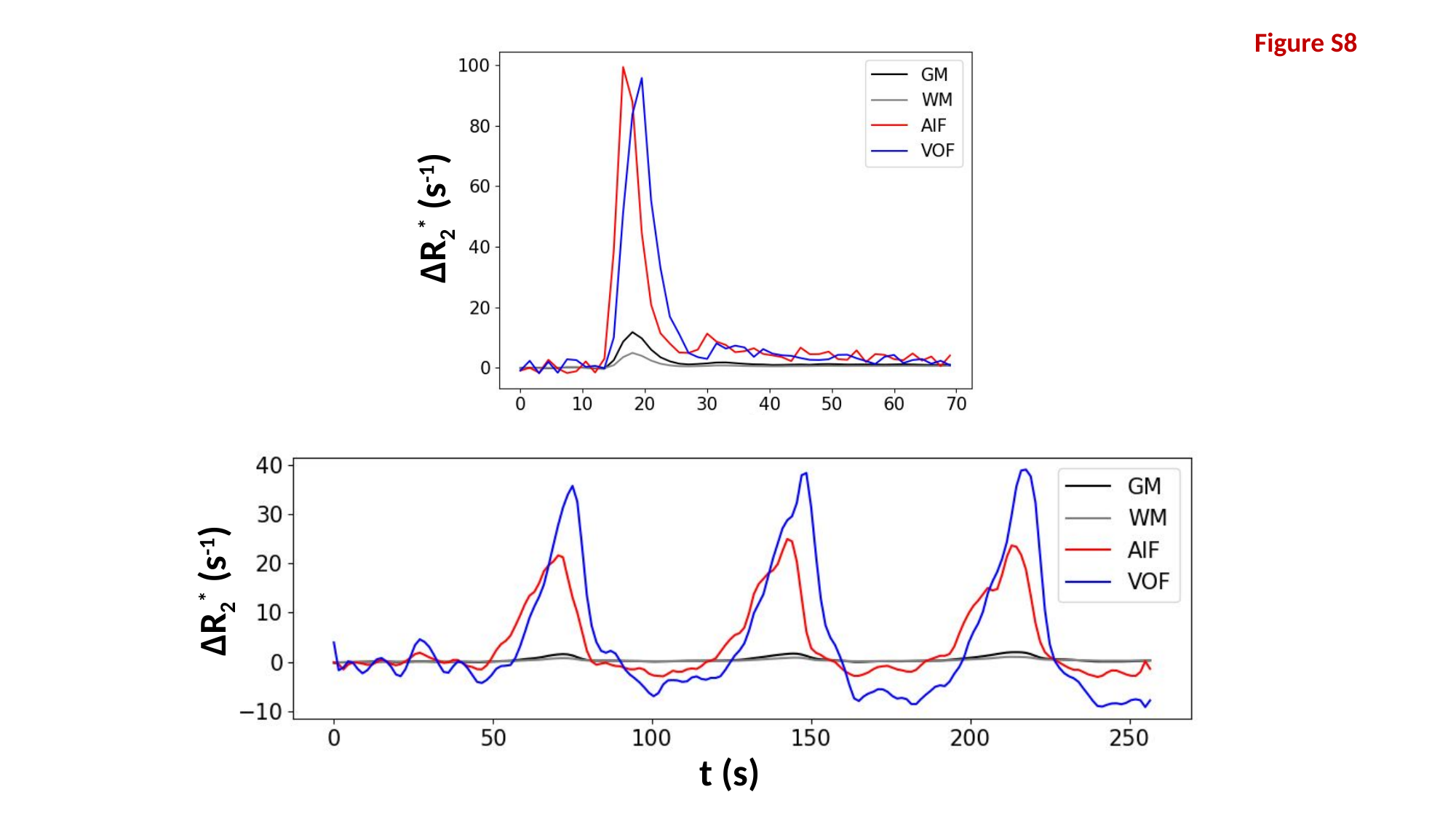

Figure S8
∆R2* (s-1)
∆R2* (s-1)
t (s)

## Slide 9
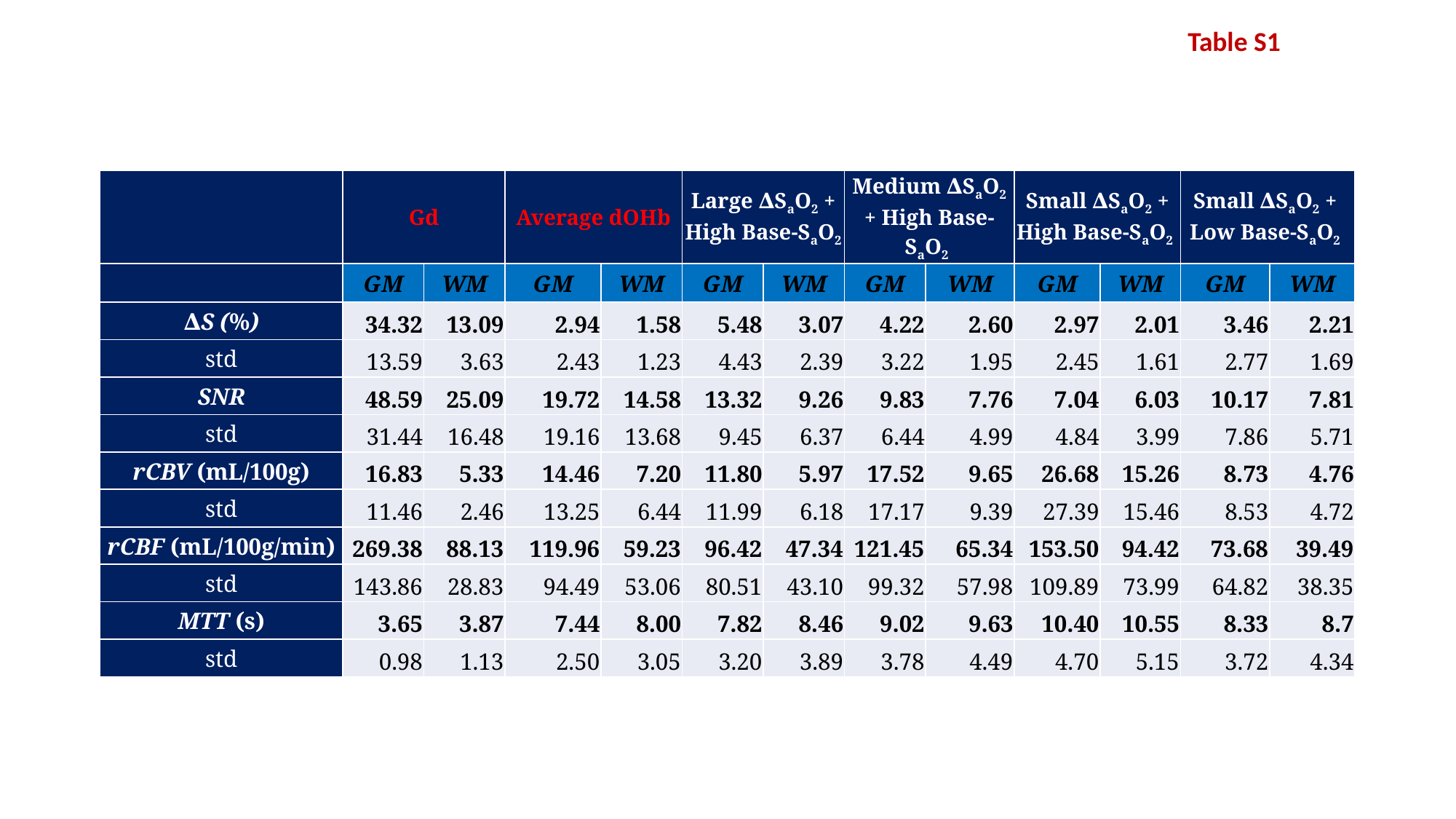

Table S1
| | Gd | | Average dOHb | | Large ∆SaO2 + High Base-SaO2 | | Medium ∆SaO2 + High Base-SaO2 | | Small ∆SaO2 + High Base-SaO2 | | Small ∆SaO2 + Low Base-SaO2 | |
| --- | --- | --- | --- | --- | --- | --- | --- | --- | --- | --- | --- | --- |
| | GM | WM | GM | WM | GM | WM | GM | WM | GM | WM | GM | WM |
| ∆S (%) | 34.32 | 13.09 | 2.94 | 1.58 | 5.48 | 3.07 | 4.22 | 2.60 | 2.97 | 2.01 | 3.46 | 2.21 |
| std | 13.59 | 3.63 | 2.43 | 1.23 | 4.43 | 2.39 | 3.22 | 1.95 | 2.45 | 1.61 | 2.77 | 1.69 |
| SNR | 48.59 | 25.09 | 19.72 | 14.58 | 13.32 | 9.26 | 9.83 | 7.76 | 7.04 | 6.03 | 10.17 | 7.81 |
| std | 31.44 | 16.48 | 19.16 | 13.68 | 9.45 | 6.37 | 6.44 | 4.99 | 4.84 | 3.99 | 7.86 | 5.71 |
| rCBV (mL/100g) | 16.83 | 5.33 | 14.46 | 7.20 | 11.80 | 5.97 | 17.52 | 9.65 | 26.68 | 15.26 | 8.73 | 4.76 |
| std | 11.46 | 2.46 | 13.25 | 6.44 | 11.99 | 6.18 | 17.17 | 9.39 | 27.39 | 15.46 | 8.53 | 4.72 |
| rCBF (mL/100g/min) | 269.38 | 88.13 | 119.96 | 59.23 | 96.42 | 47.34 | 121.45 | 65.34 | 153.50 | 94.42 | 73.68 | 39.49 |
| std | 143.86 | 28.83 | 94.49 | 53.06 | 80.51 | 43.10 | 99.32 | 57.98 | 109.89 | 73.99 | 64.82 | 38.35 |
| MTT (s) | 3.65 | 3.87 | 7.44 | 8.00 | 7.82 | 8.46 | 9.02 | 9.63 | 10.40 | 10.55 | 8.33 | 8.7 |
| std | 0.98 | 1.13 | 2.50 | 3.05 | 3.20 | 3.89 | 3.78 | 4.49 | 4.70 | 5.15 | 3.72 | 4.34 |

## Slide 10
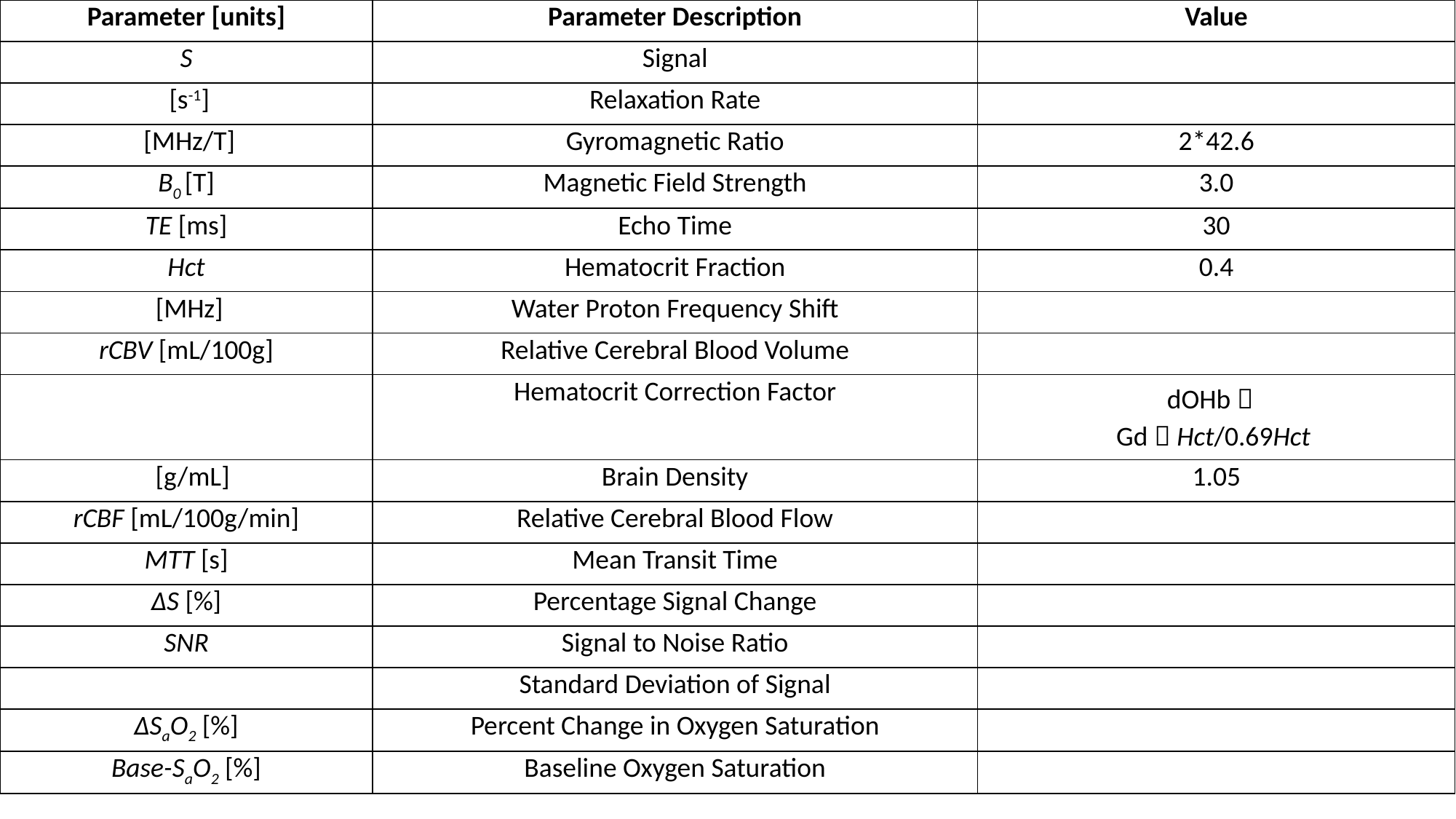

Table S2
